# Investigation of Specificity Determinants in Bacterial tRNA-Guanine Transglycosylase Reveals Queuine, the Substrate of Its Eucaryotic Counterpart, as Inhibitor

**DOI:** 10.1371/journal.pone.0064240

**Published:** 2013-05-21

**Authors:** Inna Biela, Naomi Tidten-Luksch, Florian Immekus, Serghei Glinca, Tran Xuan Phong Nguyen, Hans-Dieter Gerber, Andreas Heine, Gerhard Klebe, Klaus Reuter

**Affiliations:** Institut für Pharmazeutische Chemie, Philipps-Universität Marburg, Marburg, Germany; Max-Planck-Institute for Terrestrial Microbiology, Germany

## Abstract

Bacterial tRNA-guanine transglycosylase (Tgt) catalyses the exchange of the genetically encoded guanine at the wobble position of tRNAs^His,Tyr,Asp,Asn^ by the premodified base preQ_1_, which is further converted to queuine at the tRNA level. As eucaryotes are not able to synthesise queuine *de novo* but acquire it through their diet, eucaryotic Tgt directly inserts the hypermodified base into the wobble position of the tRNAs mentioned above. Bacterial Tgt is required for the efficient pathogenicity of *Shigella* sp, the causative agent of bacillary dysentery and, hence, it constitutes a putative target for the rational design of *anti*-Shigellosis compounds. Since mammalian Tgt is known to be indirectly essential to the conversion of phenylalanine to tyrosine, it is necessary to create substances which only inhibit bacterial but not eucaryotic Tgt. Therefore, it seems of utmost importance to study selectivity-determining features within both types of proteins. Homology models of *Caenorhabditis elegans* Tgt and human Tgt suggest that the replacement of Cys158 and Val233 in bacterial Tgt (*Zymomonas mobilis* Tgt numbering) by valine and accordingly glycine in eucaryotic Tgt largely accounts for the different substrate specificities. In the present study we have created mutated variants of *Z. mobilis* Tgt in order to investigate the impact of a Cys158Val and a Val233Gly exchange on catalytic activity and substrate specificity. Using enzyme kinetics and X-ray crystallography, we gained evidence that the Cys158Val mutation reduces the affinity to preQ_1_ while leaving the affinity to guanine unaffected. The Val233Gly exchange leads to an enlarged substrate binding pocket, that is necessary to accommodate queuine in a conformation compatible with the intermediately covalently bound tRNA molecule. Contrary to our expectations, we found that *a priori* queuine is recognised by the binding pocket of bacterial Tgt without, however, being used as a substrate.

## Introduction

**Figure 1:**
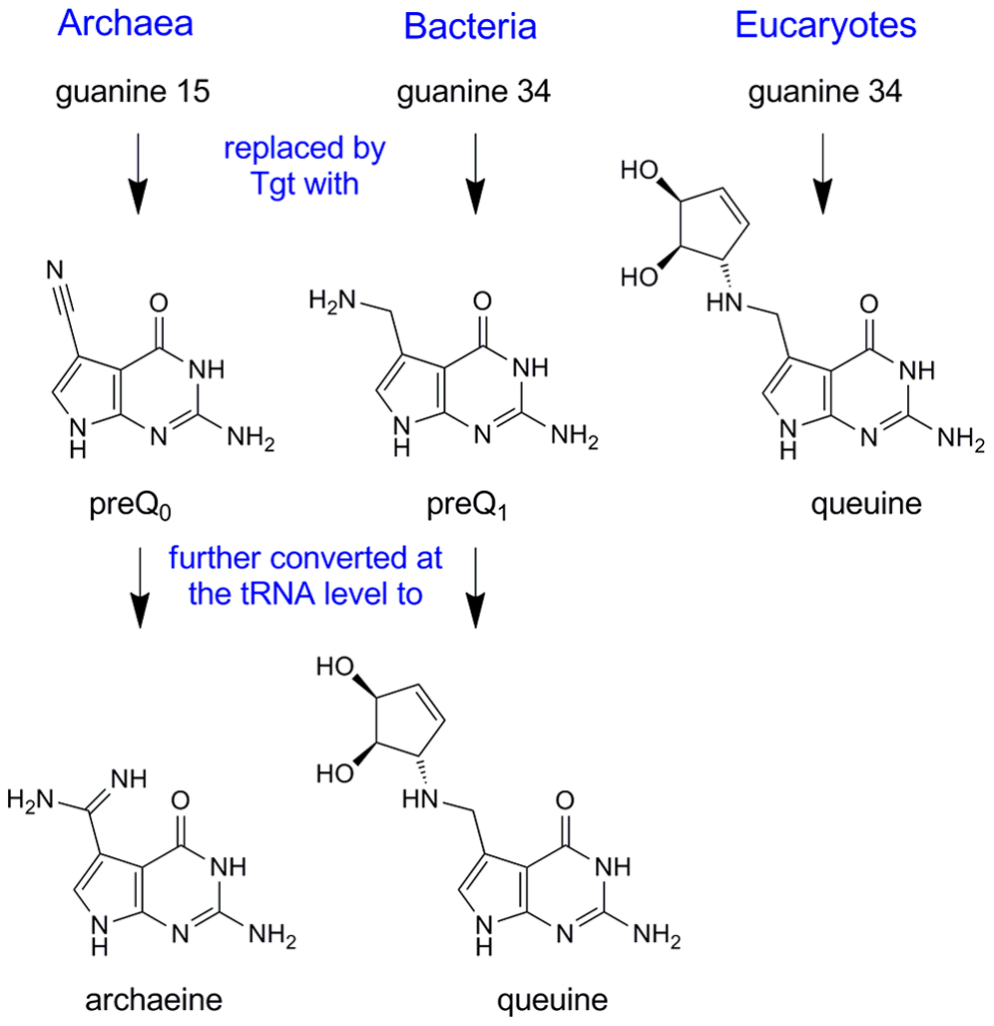
Substrate bases of the Tgt enzymes from the three domains of life. preQ_0_, 7-cyano-7-deazaguanine; preQ_1_, 7-aminomethyl-7-deazaguanine; queuine = 7-(((4,5-*cis*-dihydroxy-2-cyclopenten-1-yl) amino) methyl)-7-deazaguanine.

Transfer RNA–guanine transglycosylase (Tgt, EC 2.4.2.29) catalyses the exchange of a specific guanine base in tRNA molecules by a substituted 7-deazaguanine. Although Tgt is found in all three domains of life, the specificity of this enzyme with respect to (i) the tRNA substrate, (ii) the position of the guanine base therein to be exchanged, and (iii) the 7-deazaguanine derivative to be inserted differs in archaea, bacteria and eucaryotes (for review see [1]). Thus, the archaeal Tgt inserts preQ_0_ at position 15 of the majority of archaeal tRNAs where it is further converted to archaeine (for chemical formulae see Figure 1). Located at the “elbow” of the L-shaped tRNA the modification stabilises the overall tRNA structure by supporting the formation of the non-canonical Levitt base pair with cytosine 48 [2].

In contrast, both bacterial and eucaryotic Tgts are required for the introduction of the hypermodified base queuine into position 34 (the anticodon “wobble position”) of tRNAs^His,Tyr,Asp,Asn^ all of which have a uracil 33 – guanine 34 – uracil 35 sequence in common [3], [4]. Although no distinct function for queuine has been demonstrated yet, its presence within the tRNA anticodon suggests that it may be involved in coordinating translational fidelity and speed.

**Figure 2:**
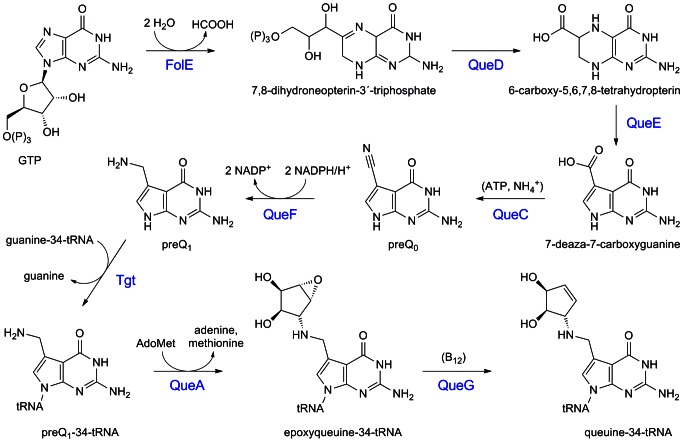
Biosynthesis of the modified tRNA base queuine. Substrates and cosubstrates: GTP, guanosine 5′-triphosphate; AdoMet, *S*-adenosylmethionine; B_12_, coenzyme B_12_; preQ_0_, 7-cyano-7-deazaguanine; preQ_1_, 7-aminomethyl-7-deazaguanine; enzymes: FolE, GTP cyclohydrolase I; QueD, 6-carboxy-5,6,7,8-tetrahydropterin synthase; QueE, 7-carboxy-7-deazaguanine synthase; QueC, preQ_0_ synthetase; QueF, preQ_0_ reductase; Tgt, tRNA-guanine transglycosylase; QueA, *S*-adenosylmethionine:tRNA ribosyltransferase-isomerase; QueG, epoxyqueuosine reductase.

Bacterial Tgt replaces the genetically encoded guanine at the above-mentioned position by the queuine-precursor preQ_1_. This premodified base is synthesised from guanosine 5′-triphosphate in a pathway employing GTP cyclohydrolase I (FolE) [5], 6-carboxy-5,6,7,8-tetrahydropterin synthase (QueD) [6], an *S*-adenosyl-l-methionine-dependent organic radical-generating enzyme (QueE), [7], preQ_0_ synthetase (QueC = ToyM) [7] and a nitrile reductase (QueF) [8]–[10]. Once incorporated into tRNA, preQ_1_ is further converted to the functional queuine in two consecutive steps catalysed by *S*-adenosylmethionine:tRNA ribosyltransferase-isomerase (QueA) [11]–[13] and the coenzyme B_12_-dependent epoxyqueuosine reductase (QueG) [14], [15] (Figure 2).

**Figure 3:**
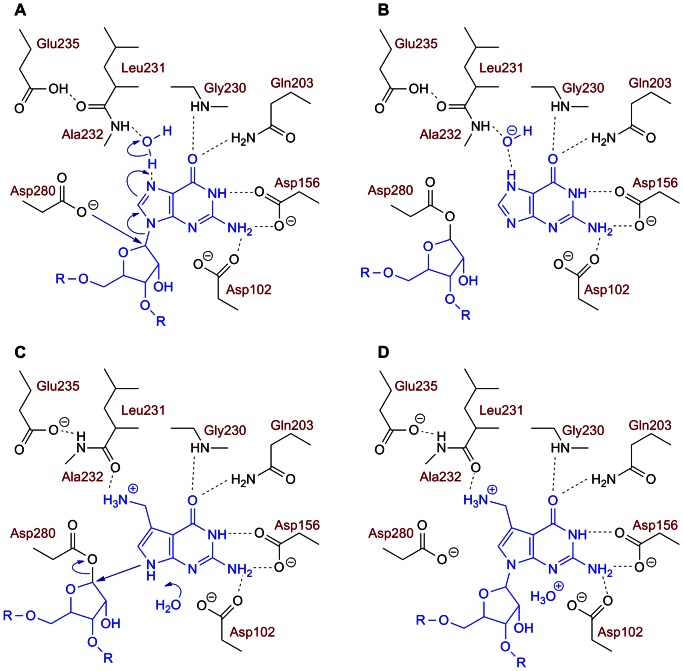
Assumed catalytic mechanism of bacterial Tgt. (A and B) The glycosidic bond of guanosine 34 is cleaved *via* nucleophilic attack by the Asp280 carboxylate resulting in the formation of a covalent Tgt⋅tRNA intermediate. (C and D) Guanine is replaced by preQ_1_ which is incorporated into the tRNA *via* nucleophilic attack of the ribose 34 anomeric carbon by *N*9 of preQ_1_. Notably the replacement of guanine by preQ_1_ in the binding pocket of Tgt induces a flip of the Leu231/Ala232 peptide bond. The formation of a hydroxide and an oxonium ion as byproducts of the reaction is assumed to be responsible for its irreversibility as mutual neutralisation will efficiently detract these ions from equilibrium. *H*-bonds are indicated by dashed lines.

Bacterial Tgt functions as a homodimer in which the protomer consists of a (βα)_8_ barrel harbouring a Zn^2+^ binding subdomain close to the *C*-terminus [16]. For sterical reasons, this dimer is, although endowed with two active sites, able to bind and convert only one substrate tRNA molecule at a time [17]. The reaction catalysed by bacterial Tgt is accurately documented by numerous biochemical studies and crystal structures of Tgt from *Zymomonas mobilis* determined in its apo-form, in the presence of guanine, preQ_1_ or small molecule inhibitors and in complex with an RNA substrate [16], [18]–[24]. The reaction follows a ping-pong mechanism including a covalent Tgt⋅tRNA intermediate (Figure 3).

Since eucaryotes are, other than bacteria, not capable of synthesising queuine *de novo* but rather acquire this modified base from their diet or from the gut flora, eucaryotic Tgt has to directly recognise and insert queuine into position 34 of tRNAs^His,Tyr,Asp,Asn^. This stands in contrast to bacterial Tgt which was reported to be unable to accept this base as a substrate [25], [26]. Unlike its bacterial counterpart, eucaryotic Tgt constitutes a *hetero*dimer with both subunits, however, being homologous to the bacterial protomer [27], [28]. Accordingly, both subunits contain the three cysteines as well as the histidine required for the coordination of the structural Zn^2+^ ion. However, only one subunit includes the residues known to be important for catalysis, among these is the active-site nucleophile, Asp280 (*Z. mobilis* Tgt numbering) [21], [24]. Obviously, eucaryotic Tgt consists of a catalytic subunit and a second subunit which most probably supports the binding and the orientation of the tRNA substrate during catalysis.

It was shown that a functional Tgt is required for efficient pathogenicity of *Shigella* bacteria which are the causative agents of bacillary dysentery. A null-mutation in the *tgt* gene leads to a strongly reduced translation of *virF*-mRNA encoding a transcriptional activator required for the expression of a large number of *Shigella* pathogenicity genes [29]. So far, the exact reason for this phenomenon is unknown, though Hurt *et al.* showed that Tgt is able to replace guanine 421 of *virF* mRNA by preQ_1_ [30]. This unusual modification of an mRNA molecule might possibly have a stimulating effect on its translation. In any case, the fact that full pathogenicity of *Shigella* sp. relies on Tgt activity encouraged us to use this enzyme as a target for the rational design of *anti*-Shigellosis compounds. Meanwhile, based on the high-resolution crystal structure of *Z. mobilis* Tgt, inhibitors which show an affinity to bacterial Tgt down to the single-digit nanomolar range have been synthesised [31]–[33].

Since a Tgt enzyme is present in humans as well, it is highly desirable to create inhibitors which preferably bind the bacterial enzyme while leaving the eucaryotic one unaffected. This seems of particular significance as recent studies performed on mice showed that a queuine deficiency or disruption of the *tgt* gene impairs the conversion of phenylalanine to tyrosine due to uncontrolled oxidation of the cofactor tetrahydrobiopterin [34]. Bearing this observation in mind, it is of utmost importance to study selectivity-determining features within both types of proteins.

Although no crystal structure of a eucaryotic Tgt has been determined yet, a homology model of the *Caenorhabditis elegans* Tgt catalytic subunit that is based on the structure of *Z. mobilis* Tgt as a template, suggests a large structural similarity of the active sites of both bacterial and eucaryotic Tgt [35]. However, in the eucaryotic enzyme, the recognition of the bulkier substrate is most probably enabled by a spatial expansion of the binding pocket. The enlargement of the binding pocket is caused by a single replacement of Val233 (*Z. mobilis* Tgt numbering) in bacterial Tgt by glycine in the eucaryotic catalytic subunit. Apart from that, the model suggests that only minor changes might be necessary in order to convert the binding pocket of bacterial Tgt such that it exhibits the specificity of the eucaryotic enzyme. Hence, in an attempt to generate a model system for the active site of human Tgt and to gain insights into the relevance of particular amino acids for substrate base selectivity, we adapted the *Z. mobilis* Tgt active site by site-directed mutagenesis in order to mimic that of the human enzyme. This paper describes the extensive analysis of mutated *Z. mobilis* Tgt variants created with this objective.

## Results

### Generation of an *in silico* homology model of the human Tgt catalytic subunit

**Figure 4:**
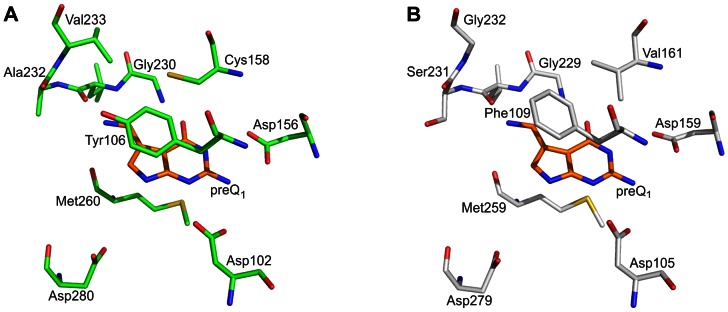
Substrate base binding pocket of *Z.* *mobilis* Tgt and modelled human Tgt.**** A) Detail of *Z. mobilis* Tgt·preQ_1_ complex crystal structure (PDB-code: **1p0e**) showing the active site with the bound substrate in stick representation. Carbon atoms of protein residues are coloured in green, those of preQ_1_ in orange. B) Homology model of human Tgt created with the *Z. mobilis* Tgt crystal structure as a template. The close up shows active site residues (carbon atoms in grey) superimposed with preQ_1_ (carbon atoms in orange) as present in **1p0e**. The coordinates of the homology model are provided within the Supporting Information (Coordinates S1).

In 2009 Boland *et al.* reported the first successful isolation and characterisation of an enzymatically active eucaryotic Tgt, which was the heterologously produced heterodimeric murine enzyme [27]. Shortly after that, Chen *et al.* published the production, purification and kinetic characterisation of recombinant human Tgt [28]. However, no crystal structure of either of these enzymes is available yet, and the only structural information concerning the active site of a eucaryotic Tgt originates from a homology model of the *Caenorhabditis elegans* catalytic subunit [35]. As the complete sequence of the human catalytic subunit has meanwhile become available [28], [36] we created a model of this orthologue using the crystal structure of *Z. mobilis* Tgt as a template (for experimental details see the “Materials and Methods” section). The model indicates that most residues establishing the active centre and substrate binding pocket in the bacterial and human enzyme are identical. A significant difference, however, is the exchange of Val233 in *Z. mobilis* Tgt by a glycine (Gly232) in human Tgt (Figure 4). The exchange leads to an enlarged binding pocket of the human enzyme, being vital to accommodate the dihydroxy-cyclopentenyl side chain of queuine which is missing in preQ_1_, the physiological substrate of the bacterial enzyme (Figure 1). A further conspicuous difference which might influence substrate selectivity is the exchange of Cys158 in *Z. mobilis* Tgt by a valine residue (Val161) in human Tgt. The side chain of Val161 in the model of human Tgt is at a position where it is likely to come in contact with the dihydroxy-cyclopentenyl moiety of a bound queuine molecule. Lastly, there is a conservative amino acid exchange within the binding pocket of the human compared to the *Z. mobilis* enzyme which will, however, most probably not affect substrate selectivity. Tyr106 within the active centre of *Z. mobilis* Tgt forms *via* its aromatic side chain a stacking interaction with the bound substrate base (see Figure 4). Not only in the human enzyme it is replaced by a phenylalanine (Phe109), but also in the majority of bacterial Tgts whose sequences are known so far. It must be noted that all of the mentioned amino acid exchanges can be observed in the model of the *C. elegans* Tgt catalytic subunit as well [35].

### Point mutations aimed at enabling *Z. mobilis* Tgt to bind queuine and kinetic characterisation of the mutated variants

In order to investigate the importance of Gly232 and Val161 in human Tgt for queuine recognition, we created a doubly mutated variant of the *Z. mobilis* enzyme in which the corresponding Val233 and Cys158 were exchanged by glycine and valine, respectively. In addition, we created two further variants, each containing solely one of the above-mentioned amino acid exchanges. To resemble the situation in the active site of human Tgt, we did not introduce the mutations into wild type *Z. mobilis* Tgt but into the Tyr106Phe variant of *Z. mobilis* Tgt. This variant had been created in the course of a former study and it had been shown that the mutation of Tyr106 to phenylalanine has no measurable impact on any of the catalytic properties of the enzyme [37]. In the following, this mutation will not be mentioned explicitly anymore and Tgt(Tyr106Phe) will henceforth be referred to as “wild type” Tgt.

To analyse the influence the introduced Val233Gly and Cys158Val mutations have on the binding of the different substrate bases and on catalysis, we measured kinetic parameters for the mutated variants and “wild type” Tgt. At first, *K*_M_ and *k*_cat_ for guanine and tRNA^Tyr^ were analysed *via* monitoring the incorporation of [8-^3^ *H*]-guanine into tRNA^Tyr^. None of the mutations had, either alone or in combination, any significant influence on *K*_M_(guanine) or *K*_M_(tRNA^Tyr^), yet *k*_cat_ had, compared to “wild type” enzyme, decreased for all three variants (see Table 1). While for Tgt(Cys158Val) the turnover number was only marginally reduced by a factor of two it was lowered by one order of magnitude for Tgt(Val233Gly). In Tgt(Cys158Val/Val233Gly) the combination of both mutations led to a decrease in *k*_cat_ by two orders of magnitude.

**Table 1:** Kinetic parameters for “wild type” Tgt and mutated Tgt variants.*

	tRNA^Tyr^ **	guanine	preQ_1_
“**Wild type**” **Tgt**			
*K*_M_ (µmol·L^−1^)	0.9±0.2	1.2±0.2	0.9±0.1
*k*_cat_ (10^−3^ s^−1^)***	53.6±0.1	55.8±0.1	106.0±2.7
**Tgt(Cys158Val)**			
*K*_M_ (µmol·L^−1^)	2.1±0.2	1.4±0.2	16.0±2.4
*k*_cat_ (10^−3^ s^−1^)***	28.1±0.3	27.5±0.2	35.0±0.8
**Tgt(Val233Gly)**			
*K*_M_ (µmol·L^−1^)	2.0±0.3	1.0±0.2	0.6±0.1
*k*_cat_ (10^−3^ s^−1^)***	5.4±0.3	4.6±0.3	6.3±0.2
**Tgt(Cys158Val/Val233Gly)**			
*K*_M_ (µmol·L^−1^)	1.4±0.4	2.7±0.3	13.5±3.1
*k*_cat_ (10^−3^ s^−1^)***	0.8±0.1	0.6±0.1	1.0±0.3

* All Tgt variants considered in this table including “wild type” Tgt contain an additional Tyr106Phe mutation which in a previous study was shown to have no impact on any of the catalytic properties of the enzyme [37] (see main text).

** Using [8-^3^ *H*]-guanine as second substrate.

*** Taking into account that Tgt is a homodimer able to bind and convert only one substrate tRNA molecule at a time [17].

Subsequently, kinetic parameters were determined for preQ_1_ by measuring the incorporation of [^3^ *H*]-preQ_1_ into tRNA^Tyr^. For Tgt(Cys158Val) also *k*_cat_(preQ_1_) was only slightly reduced by a factor of three compared to “wild type” Tgt. Remarkably, however, this mutation sparked an increase of *K*_M_(preQ_1_) by one order of magnitude, a loss of affinity which had not been observed for guanine. In contrast, no decrease in preQ_1_ affinity was detected for Tgt(Val233Gly) while, compared to “wild type” Tgt, *k*_cat_(preQ_1_) had decreased by one order of magnitude. As observed during the use of guanine as a substrate, the combination of both mutations in Tgt(Cys158Val/Val233Gly) led to a decrease in *k*_cat_(preQ_1_) by two orders of magnitude. Furthermore, *K*_M_(preQ_1_) of Tgt(Cys158Val/Val233Gly) had increased by more than a factor of ten, confirming that the Cys158Val exchange results in a measurable loss of affinity to this substrate base.

Ultimately, we intended to determine kinetic parameters for the three mutated Tgt variants as well as for “wild type” Tgt with respect to queuine. Seemingly, low activity was observed with “wild type” Tgt, Tgt(Cys158Val) and Tgt(Val233Gly) by measuring the incorporation of [^3^ *H*]-queuine into tRNA^Tyr^. Thorough analysis of the measured progress curves, however, revealed that the observed activities were obviously not due to the insertion of queuine into substrate tRNA. At queuine concentrations equal to or exceeding the concentration of tRNA^Tyr^ (15 µmol·L^−1^), the initially observed insertion of the radiolabelled base into tRNA already came to a halt when <10% of the tRNA was labelled (data not shown). This observation may be interpreted such that the used [^3^ *H*]-queuine preparation contained a [^3^ *H*]-labelled contaminant which was inserted with a measurable turnover (this contaminant might possibly be 7-deaza-7-hydroxymethylguanine, resulting from the reduction of the corresponding aldehyde which was used as starting material for queuine synthesis [26]). After the majority of this more potent impurity was incorporated into tRNA^Tyr^, queuine was barely or not inserted at all. Accordingly, this unexpected restriction did not allow us to figure out whether “wild type” Tgt, Tgt(Cys158Val) and Tgt(Val233Gly) were indeed able to accept queuine as a substrate nor did it allow us to determine Michaelis-Menten parameters as an indicator for affinity and catalytic activity. Therefore, we applied an alternative assay tracing the decrease in radioactivity of tRNA^Tyr^ labelled at position 34 with [8-^3^ *H*]-guanine due to the incorporation of unlabelled queuine. Compared to the commercially purchased [^3^ *H*]-queuine, the “cold” queuine used for this assay was produced *via* a completely different synthetic route with preQ_1_ as a starting material. NMR analysis of this queuine preparation had revealed no detectable preQ_1_ contamination implying that, if present, its portion amounted to <1‰ [38]. In fact, when queuine was used from this highly pure preparation at a concentration of 50 µmol·L^−1^ no insertion of queuine into tRNA^Tyr^ was observed for “wild type” Tgt or any of the mutated variants during a time period of four hours. In contrast, when, as a positive control, 10 µmol·L^−1^ (“cold”) preQ_1_ was used in this assay, insertion of this base into 90% of the tRNA (in this assay present at a concentration of 8 µmol·L^−1^) was achieved by “wild type” Tgt within <10 minutes (data not shown).

### Affinity of tRNA^Tyr^ to Tgt as determined by microscale thermophoresis

Since none of the Tgt variants investigated in this study showed turnover when queuine was used as a substrate it was not possible to determine *K*_M_(queuine) as a measure of affinity. Therefore, in order to gain information about the ability of these variants to bind queuine we intended to use “microscale thermophoresis” to quantify dissociation constants (*K*_D_) of the enzyme·queuine complexes. This method is based on a microscale temperature gradient which is produced in a solution within a capillary by an infrared LED laser. Proteins (as well as any other molecules) show a directional motion along the temperature gradient determined by their size, charge and hydration shell which in turn are influenced by the presence or absence of a bound ligand. Any change in the above-named parameters caused by ligand binding affects the thermophoretic movement of the investigated protein, allowing the determination of binding affinities [39]. To assess the applicability of this method to *Z. mobilis* Tgt we intended to determine *K*_D_ values for guanine, preQ_1_, queuine and tRNA^Tyr^ complexes of Tgt. However, no change in thermophoresis was observed upon addition of guanine, preQ_1_ and queuine proving this method to be unsuitable to investigate the affinities of these bases to Tgt and its mutated variants.

**Figure 5:**
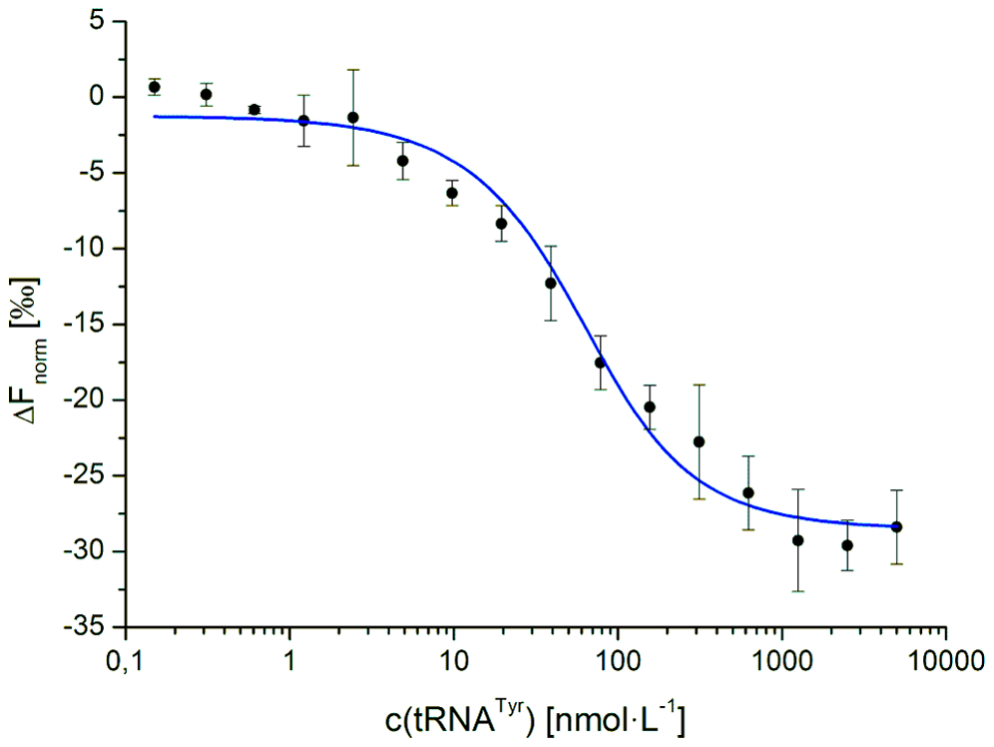
Binding of tRNA^Tyr^ to Tgt measured by Microscale Thermophoresis. Alexa Fluor®647 labelled Tgt at a concentration of 50 nmol·L^−1^ was incubated with varying amounts of tRNA^Tyr^. The difference in normalised fluorescence as a measure of change in thermophoresis is plotted against the concentration of tRNA^Tyr^.

In contrast, a strong influence on the thermophoretic behaviour of Tgt was noticed upon binding of the macromolecular substrate tRNA^Tyr^ enabling the determination of *K*_D_(Tgt·tRNA^Tyr^) which amounted to 36.2 nmol·L^−1^±5.7 nmol·L^−1^ (Figure 5). This value is considerably lower than *K*_M_(tRNA^Tyr^) (Table 1) indicating an affinity of Tgt to this substrate which is clearly higher than so far assessed by enzyme kinetics studies. The reason for this discrepancy may be that the binding of guanine to apo-Tgt prevents the binding of tRNA^Tyr^ to the enzyme. Thus, guanine not only acts as a substrate but also as a competitive inhibitor with respect to tRNA. As guanine is present in a saturating concentration in any assay that aims at determining *K*_M_(tRNA^Tyr^) of Tgt, the recorded value may not constitute a reliable measure but will rather lead to a significant underestimation of the enzyme's affinity to tRNA^Tyr^.

### Inhibition of Tgt and its mutated variants by queuine

Since queuine is not used as a substrate by “wild type” Tgt and our mutated variants, any specific binding of this base to the active centres of these enzymes should inevitably cause their inhibition. To verify an inhibitory effect of queuine on Tgt activity, we measured the activity of “wild type” Tgt, Tgt(Cys158Val), Tgt(Val233Gly) and Tgt(Cys158Val/Val233Gly) using guanine and tRNA^Tyr^ as substrates both in the presence and absence of a low µmol·L^−1^ concentration of queuine. Indeed, a considerable decrease in acitivity dependent on queuine was observed for all investigated Tgt variants (see below).

As inhibitors mimicking the nucleobases guanine or preQ_1_ are basically able to inhibit Tgt in two different ways, we next determined the inhibition mode *via* that queuine acts on the investigated enzyme variants. If an inhibitor binds to the guanine 34/preQ_1_ 34 subpocket of the apo-enzyme, it will inhibit the tRNA from accessing the enzyme and thus it will act as a competitive inhibitor with respect to the tRNA substrate. If, however, such an inhibitor occupies this subpocket after tRNA binding and the removal of guanine 34 it will prevent the substrate base preQ_1_ from binding. Accordingly, any further reaction of the covalent Tgt·tRNA intermediate is disabled which corresponds to an uncompetitive inhibition with respect to the tRNA substrate. Nucleobase mimetics of comparable size as guanine or preQ_1_ normally act *via* both inhibition modes. Nucleobase mimetics endowed with bulky exocyclic substituents, however, generally constitute purely competitive inhibitors with respect to tRNA as their binding to the covalent Tgt·tRNA complex would sterically interfere with the tRNA sugar phosphate backbone.

To figure out the inhibition mode *via* that queuine acts on the investigated Tgt variants we applied a trapping experiment which had been used for a similar purpose by Meyer *et al.* [23]. Incubation of Tgt with substrate tRNA in the presence of an uncompetitive inhibitor in excess leads to a considerable stabilisation of the covalent Tgt·tRNA intermediate which can be visualised in terms of two retarded bands on a Coomassie-stained gel after SDS-PAGE of the reaction mixture (the presence of two retarded bands representing this complex results from different tRNA conformers present under the applied PAGE conditions [40]). In the absence of any inhibitor, only a very small amount of the covalent intermediate is detectable under the used assay conditions. The presence of excess inhibitor which solely and efficiently acts as a competitive inhibitor with respect to substrate tRNA entirely suppresses the formation of the covalent intermediate.

**Figure 6:**
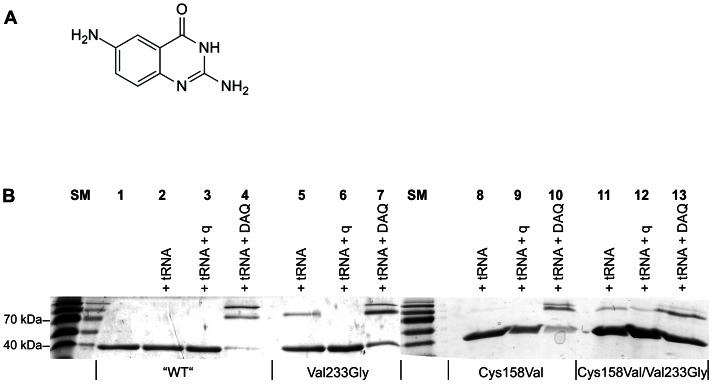
Trapping experiments performed with Tgt/tRNA mixtures in presence of queuine or 2,6-diamino-3*H*-quinazolin-4-one. A) Chemical structure of 2,6-diamino-3*H*-quinazolin-4-one (DAQ), an uncompetitive inhibitor of Tgt. B) SDS-PAGE analysis of reaction mixtures of Tgt or mutated variants thereof and tRNA^Tyr^ under conditions indicated. SM, size marker; q, queuine. While DAQ causes retarded Tgt bands by stabilising the covalent Tgt·tRNA intermediate, queuine lacks this ability for most of the investigated Tgt variants. Solely in case of Tgt(Cys159Val/Val233Gly) a faint retarded band is visible indicating that queuine may to some extent be able to bind to the guanine 34/preQ_1_ subpocket of the covalent enzyme·tRNA complex.

Thus, we incubated “wild type” Tgt, Tgt(Cys158Val), Tgt(Val233Gly) and Tgt(Cys158Val/Val233Gly) with tRNA^Tyr^ in the presence of excess queuine. As a control, we incubated each of these Tgt variants solely with tRNA^Tyr^ as well as with tRNA^Tyr^ plus an excess of the preQ_1_ mimetic 2,6-diamino-3*H*-quinazolin-4-one (DAQ) (Figure 6A) which constitutes a potent uncompetitive inhibitor of Tgt (*K*_iu_ = 0.6 µmol·L^−1^) [23]. Subsequently, we subjected the reaction mixtures to SDS-PAGE. In the case of “wild type” Tgt and Tgt(Cys158Val) no retarded band representing the covalent Tgt·tRNA intermediate was detectable on the Coomassie-stained gel, when, in addition to the enzyme, only tRNA^Tyr^ was present in the reaction mixture (Figure 6B, lanes 2 and 8). This indicates that any excised guanine was quickly reinserted into tRNA keeping the amount of the covalent complex below the detection limit of the assay. In contrast, under the same conditions a faint band representing the covalent enzyme·tRNA complex was observed for Tgt(Val233Gly) and Tgt(Cys158Val/Val233Gly) indicating that the reinsertion rate of the excised guanine was significantly decreased with these Tgt variants (Figure 6B, lanes 5 and 11). When, in addition to tRNA^Tyr^ excess DAQ was added, in the case of all Tgt variants the majority of the enzyme was trapped in a covalent complex formed with tRNA^Tyr^. On the Coomassie-stained gel this complex was visible as two clear retarded bands concomitant with considerable weakening of the band corresponding to the uncomplexed enzyme (Figure 6B, lanes 4, 7, 10 and 13). When queuine, instead of DAQ, was added to the reaction mixture, the formation of the covalent intermediate was completely suppressed in the case of “wild type” Tgt, Tgt(Cys158Val) and Tgt(Val233Gly) (Figure 6B, lanes 3, 6 and 9). This suggests that for these Tgt variants, queuine predominantly acts as a competitive inhibitor with respect to substrate tRNA. Solely in the case of Tgt(Cys158Val/Val233Gly) a very faint band representing the covalent enzyme·tRNA complex was visible in the presence of excess queuine (Figure 6B, lane 12). This may indicate that, in the case of this mutated variant, queuine is to some extent able to bind to the guanine 34/preQ_1_ subpocket of the covalent enzyme·tRNA complex resulting in an inhibition uncompetitive with tRNA binding. The fact, however, that the band representing the covalent enzyme·tRNA complex is clearly weaker than that observed in the absence of queuine suggests that queuine will also mainly bind to this mutated variant when present in the apo-form.

To gain information concerning the affinity of queuine to “wild type” Tgt and the mutated variants we determined, according to the method described in [23], the corresponding competitive inhibition constants, with respect to tRNA (*K*_ic_), of queuine. *K*_ic_(queuine) amounted to 3.8 µmol·L^−1^±0.6 µmol·L^−1^ for “wild type” Tgt, 3.8 µmol·L^−1^±0.5 µmol·L^−1^ for Tgt(Cys158Val), 3.1 µmol·L^−1^±0.2 µmol·L^−1^ for Tgt(Val233Gly) and 8.9 µmol·L^−1^±1.2 µmol·L^−1^ for Tgt(Cys158Val/Val233Gly). These results show that all studied Tgt variants including “wild type” Tgt are able to bind queuine with nearly identical affinity.

### Crystal structure analyses of “wild type” Tgt and its mutated variants

To allow the interpretation of the obtained data at a structural level, we determined the crystal structures of the mutated Tgt variants created in this study in their apo-forms as well as in complex with preQ_1_ and queuine. In addition, we determined the crystal structure of “wild type” Tgt with a bound queuine molecule. Structures of “wild type” Tgt in its apo-form and in complex with preQ_1_ had already been published in a former study [37] and thus, were available in the protein data bank (PDB codes: **1ozm** and **1ozq**, respectively). An overview of the crystal structures considered in the present study is given in Table S1, while detailed data statistics are presented in Tables 2, 3 and 4.

**Table 2:** Crystallographic data collection and refinement statistics - Tgt(Cys158Val).

Crystal data*	Tgt(Cys158Val)	Tgt(Cys158Val)·guanine	Tgt(Cys158Val)·preQ_1_	Tgt(Cys158Val)·queuine
PDB ID	4gd0	4h7z	4e2v	4hvx
**A. Data Collection and Processing**				
Beamline	BL 14.2	BL 14.1	BL 14.2	BL 14.2
λ (Å)	0.91841	0.91841	0.91841	0.91841
Space group	*C*2	*C2*	*C*2	*C*2
*a*, *b*, *c* (Å)	91.4, 65.0, 70.2	90.4, 64,8, 70,8	90.4, 64.9, 70.4	91.6, 64.5, 71.1
β (°)	96.1	95.6	95.8	96.2
Matthews coefficient (Å^3^/Da)	2.4	2.4	2.4	2.4
Solvent content (%)	48.7	48.7	48.7	48.7
**B. Diffraction Data**				
Resolution range^a^ (Å)	50-1.29 (1.31-1.29)	30-1.68 (1.77-1.68)	21-1.18 (1.2-1.18)	50-1.82 (1.85-1.82)
No. of unique reflections	102,682 (4187)	45,941 (6706)	132,461 (5750)	35405 (1467)
Completeness^a^ (%)	97.1 (81.5)	99.2 (99.8)	95.0 (88.0)	94.9 (78.4)
Redundancy	2.7 (1.7)	2.9 (2.8)	2.5 (2.2)	3.0 (2.2)
R(I)_sym_ a *^,^* b (%)	4.7 (23.3)	7.0 (48.2)	4.2 (21.8)	4.5(36.3)
I/σ(I)^a^	22.1 (3.0)	10.2 (2.1)	21.4 (4.1)	21.7 (2.2)
**C. Refinement**				
Program	Phenix	Phenix	Phenix	Phenix
*R*_work_ c/*R*_free_ d (%)	16.2/18.5	15.7/19.1	13.7/15.2	16.6/19.7
Protein residues	373	371	364	358
Water molecules	524	318	464	165
Ligand atoms	—	11	13	13
Ramachandran plot				
Residues in most favored regions (%)	94.4	95.5	95.3	95.4
Residues in additionally allowed regions (%)	5.0	4.2	4.4	4.3
Residues in generously allowed regions (%)	0.6	0.3	0.3	0.3
Mean *B*-factors (Å^2^)				
Protein	12.6	15.6	13.7	28.6
Water	29.1	29.4	29.2	35.8
Ligand	—	29.8	11.6	34.1
Rmsd from ideality				
rmsd angle (°)	1.1	1.8	1.2	1.2
rmsd bond (Å)	0.005	0.019	0.007	0.010

* All Tgt variants considered in this table contain an additional Tyr106Phe mutation which in a previous study was shown to have no impact on any of the catalytic properties of the enzyme [37] (see main text).

a ) number in parentheses is for highest resolution shell.

b ) 
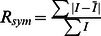
, with *I* representing the observed intensity and *Ī* representing the average intensities for multiple measurements.

c ) 
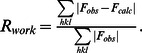

d ) *R*_free_ was calculated as *R*_work_ but on 5% of the data excluded from the refinement.

**Table 3:** Crystallographic data collection and refinement statistics – Tgt(Val233Gly).

Crystal data*	Tgt(Val233Gly)	Tgt(Val233Gly)·preQ_1_	Tgt(Val233Gly)·queuine
PDB ID	3bl3	3bld	4hsh
**A. Data Collection and Processing**			
Beamline	In-house	BL 14.2	BL 14.2
λ (Å)	1.54178	0.91841	0.91841
Space group	*C*2	*C*2	*C*2
*a*, *b*, *c* (Å)	90.7, 64.8, 70.4	89.7, 64.9, 70.3	90.5, 64.8, 70.5
β (°)	95.9	95.6	95.9
Matthews coefficient (Å^3^/Da)	2.4	2.4	2.4
Solvent content (%)	48.7	48.7	48.7
**B. Diffraction Data**			
Resolution range^a^ (Å)	50-2.25 (2.30-2.25)	50-1.19 (1.22 -1.19)	50-1.56 (1.59-1.56)
No. of unique reflections	49,829	116,963	57,783
Completeness^a^ (%)	93.4 (66.4)	91.6 (62.8)	100.0 (100.0)
Redundancy	2.8	3.7	4.2 (4.1)
R(I)_sym_ a *^,^* b (%)	11.0 (28.1)	4.1 (26.2)	8.8 (49.6)
I/σ(I)^a^	9.0 (2.4)	21.0 (2.7)	15.4 (2.7)
**C. Refinement**			
Programm	ShelxL-97	ShelxL-97	Phenix
*R*_work_ c/*R*_free_ d (%)	18.7/22.7	15.1/18.5	15.5/18.1
Protein residues	365	348	365
Water molecules	216	262	318
Ligand atoms	-	13/1	12/1
Ramachandran plot			
Residues in most favored regions (%)	94.9	95.6	95.5
Residues in additionally allowed regions (%)	4.8	4.1	4.2
Residues in generously allowed regions (%)	0.3	0.3	0.3
Mean *B*-factors (Å^2^)			
Protein	19.9	18.6	16.8
Water	28.4	28.4	31.9
Ligand	—	33.0	36.0
RMSD from ideality			
rmsd angle (°)	1.3	2.1	1.6
rmsd bond (Å)	0.008	0.013	0.017

* All Tgt variants considered in this table contain an additional Tyr106Phe mutation which in a previous study was shown to have no impact on any of the catalytic properties of the enzyme [37] (see main text).

a ) number in parentheses is for highest resolution shell.

b ) 
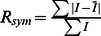
, with *I* representing the observed intensity and *Ī* representing the average intensities for multiple measurements.

c ) 
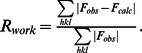

d ) *R*_free_ was calculated as *R*_work_ but on 5% of the data excluded from the refinement.

**Table 4:** Crystallographic data collection and refinement statistics – Tgt(Cys158Val/Val233Gly), “WT”.

Crystal data*	Tgt(Cys158Val/Val233Gly)	Tgt(Cys158Val/Val233Gly)·preQ_1_	Tgt(Cys158Val/Val233Gly)·queuine	“WT”-Tgt·queuine
PDB ID	4h6e	4gcx	4hqv	**
**A. Data Collection and Processing**				
Beamline	BL 14.2	BL 14.2	BL 14.2	BL 14.2
λ (Å)	0.91841	0.91841	0.91841	0.91841
Space group	*C2*	*C2*	*C2*	*C2*
*a*, *b*, *c* (Å)	91.0, 64.8, 70.5	91.2, 65.1, 70.6	90.9, 65.0, 70.6	90.8, 64.7, 70.6
β (°)	96.1	96.1	96.3	95.9
Matthews coefficient (Å^3^/Da)	2.4	2.4	2.4	2.4
Solvent content (%)	48.7	48.7	48.7	48.7
**B Diffraction Data**				
Resolution range^a^ (Å)	30-1.42 (1.44-1.42)	50-1.42 (1.44-1.42)	50-1.66 (1.69-1.66)	50-1.22(1.24-1.22)
No. of unique reflections	76,896 (3,342)	77,486 (3,750)	48,267 (2,279)	117,888 (4,937)
Completeness^a^ (%)	96.7 (87.2)	99.0 (96.2)	97.3 (95.5)	97.3 (81.7)
Redundancy	2.8 (2.1)	3.1 (2.6)	3.0 (2.9)	4.0 (3.3)
R(I)_sym_ a *^,^* b (%)	4.3 (15.5)	6.7 (41.6)	9.7 (47.3)	4.6 (41,7)
I/σ(I)^a^	22.9 (4.6)	16.5 (2.2)	10.7 (2.5)	26.1 (2.6)
**C. Refinement**				
Programm	Phenix	Phenix	Phenix	Phenix
*R*_work_ c/*R*_free_ d (%)	14.9/17.4	13.1/16.1	17.5/20.7	13.6/15.7
Protein residues	272	369	273	371
Water molecules	421	397	397	334
Ligand atoms	—	13	13	—
Ramachandran plot				
Residues in most favored regions (%)	95.5	94.9	94.7	95.9
Residues in additionally allowed regions (%)	4.7	4.8	4.7	3.8
Residues in generously allowed regions (%)	0.3	0.3	0.6	0.3
Mean *B*-factors (Å^2^)				
Protein	8.3	14.2	17.7	16.4
Water	22.8	32.5	29.7	30.0
Ligand	—	15.7	30.0	—
RMSD from ideality				
rmsd angle (°)	1.4	1.1	1.1	1.6
rmsd bond (Å)	0.010	0.006	0.007	0.014

* All Tgt variants considered in this table including “wild type” Tgt contain an additional Tyr106Phe mutation which in a previous study was shown to have no impact on any of the catalytic properties of the enzyme [37] (see main text).

** As the refined model contains no ligand the coordinates and structure factors were not deposited with the Protein Data Base but are provided within the Supporting Information (Coordinates S2; Structure factors S1).

a ) number in parentheses is for highest resolution shell.

b ) 
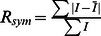
, with *I* representing the observed intensity and *Ī* representing the average intensities for multiple measurements.

c ) 
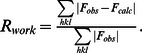

d ) *R*_free_ was calculated as *R*_work_ but on 5% of the data excluded from the refinement.

The 2|F_o_|-|F_c_| electron density maps of all apo-structures clearly allow the identification of the introduced mutations, although a rather poorly defined density is present for Gly233 in both the structures of Tgt(Val233Gly) and Tgt(Cys158Val/Val233Gly). In the electron density maps of Tgt(Cys158Val) and of Tgt(Cys158Val/Val233Gly) the isopropyl side chain of Val158 is excellently defined. This is contrary to the sulfanylmethyl group of the original Cys158 whose side chain is, obviously due to its flexibility, poorly defined in the electron density maps of virtually all *Z. mobilis* Tgt crystal structures determined so far. Superimposition of the apo-structures of the mutated variants and “wild type” Tgt revealed that none of the amino acid exchanges implicates any significant structural change concerning the remaining part of the protein (mean rmsd 0.23 Å±0.02 Å).

**Figure 7:**
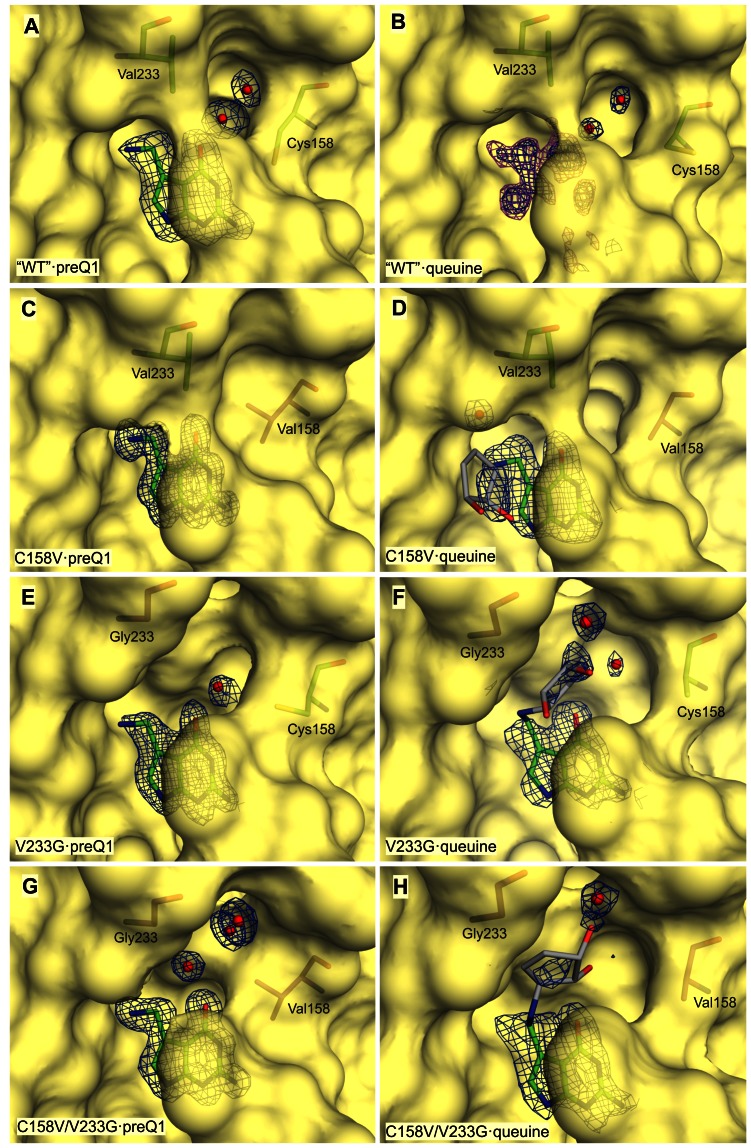
Crystal structures of Tgt variants in complex with preQ_1_ or queuine: surface representation of substrate pockets. The solvent accessible surfaces of active sites from *Z. mobilis* Tgt variants are shown in bright yellow. The respective Tgt variant plus the bound ligand (shown in stick representation) are indicated in each sub-figure. Also amino acid residues at positions 158 and 233 are shown in stick representation. Carbon atoms of original amino acids and of the bound ligand are coloured green, those of mutated amino acids magenta. As the electron density assignable to the dihydroxy-cyclopentenyl moiety of queuine is poorly defined in all structures containing this ligand the coordinates of this moiety are not present in the respective structures deposited with the Protein Data Base. Accordingly, the conformations of the dihydroxy-cyclopentenyl shown in (D), (F) and (H) are tentative. To indicate this fact, the carbon atoms of this moiety are shown in grey. Selected water molecules are shown as red spheres. 2|F_o_|-|F_c_| (at σ 1.0) electron density is shown for the bound ligand and water molecules. 2|F_o_|-|F_c_| electron density contoured at a σ level of 1.0 is coloured blue, |F_o_|-|F_c_| electron density contoured at a σ level of 2.5 is coloured magenta. An overview of the crystal structures analysed in this study including resolutions and PDB codes is given in Table S1.

**Figure 8:**
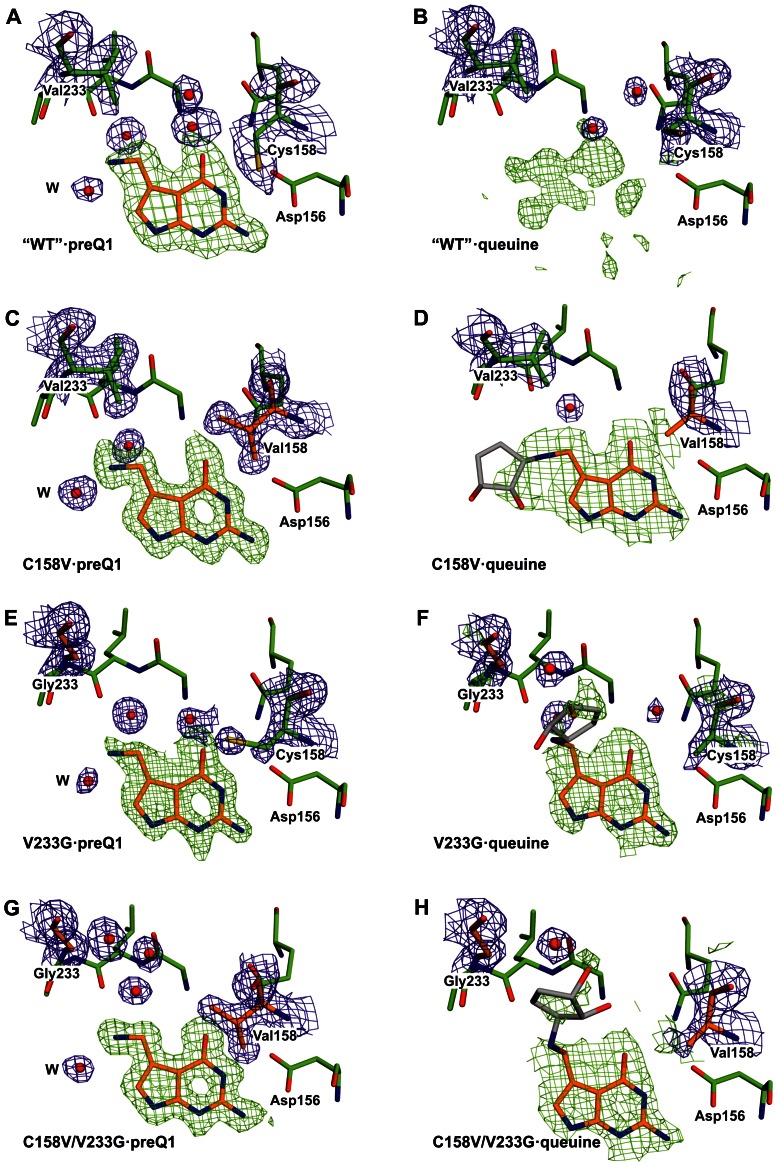
Crystal structures of Tgt variants in complex with preQ_1_ or queuine: stick representation of substrate pockets. The respective Tgt variant plus the bound ligand are indicated in each sub-figure. Carbon atoms of original amino acids are coloured green, those of mutated amino acids as well as of the bound ligand orange. As the electron density assignable to the dihydroxy-cyclopentenyl moiety of queuine is poorly defined in all structures containing this ligand the coordinates of this moiety are not present in the respective structures deposited with the Protein Data Base. Accordingly, the conformations of the dihydroxy-cyclopentenyl shown in (D), (F) and (H) are tentative. To indicate this fact, the carbon atoms of this moiety are shown in grey. Selected water molecules are shown as red spheres. Electron density is shown for amino acid residues at positions 158 and 233 as well as for the ligand and for water molecules. The 2|F_o_|-|F_c_| electron density map contoured at a σ level of 1.0 is coloured blue. The green density represents an |F_o_|-|F_c_| omit map contoured at 2.5 σ. An overview of the crystal structures analysed in this study including nominal resolutions and PDB codes is given in Table S1.

Next, we analysed and compared the structures of the studied Tgt variants in complex with preQ_1_. In all preQ_1_-bound structures the ligand appears well-defined in the respective electron density map. Superimposition of the structural models reveals that preQ_1_ is identically positioned in all investigated variants including “wild type” Tgt (mean rmsd = 0.20 Å±0.07 Å). Furthermore, no differences were observed with respect to the catalytic Asp280 and the conformations of amino acids known to form direct interactions with the bound preQ_1_ ligand. The most conspicuous differences among the preQ_1_-bound structures concern a small cavity separating the side chains of amino acid residues 158 and 233 (Figure 7). In “wild type” Tgt this cavity harbors two well-ordered water molecules (Figure 7A) which are displaced in the Tgt(Cys158Val)·preQ_1_ complex structure by the isopropyl side chain of Val158 (Figure 7C). In the structure of preQ_1_-bound Tgt(Val233Gly) this cavity is substantially widened but harbors only one well-ordered water molecule. In addition, the electron density assigned to the preQ_1_ ligand does obviously not reflect the excellent resolution of this structure (1.19 Å) (Figures 7E and 8E). This is also indicated by the mean *B*-factor of preQ_1_ which is, in this structure, considerably elevated compared to the mean *B*-factor of the protein (33.0 Å^2^ *versus* 18.6 Å^2^). In contrast, in the structures of all other preQ_1_-bound Tgt variants the mean *B*-factor of preQ_1_ deviates only slightly from the mean *B*-factor of the protein (see Tables 2 and 4). The structure of preQ_1_-bound Tgt(Cys158Val/Val233Gly) reveals that, compared to Tgt(Val233Gly), the replacement of the hydrophilic and flexible sulfanylmethyl moiety of Cys158 by the hydrophobic isopropyl of valine leads to a regeneration of the original order in the substrate base binding pocket. The mean *B*-factor of the bound preQ_1_, which is excellently defined in the electron density map, hardly differs from the mean *B*-factor of the protein (15.7 Å^2^ *versus* 14.2 Å^2^). Furthermore, an arrangement of three highly ordered water molecules is present within the spacious cavity between Val158 and Gly233 (Figure 7G).

**Figure 9:**
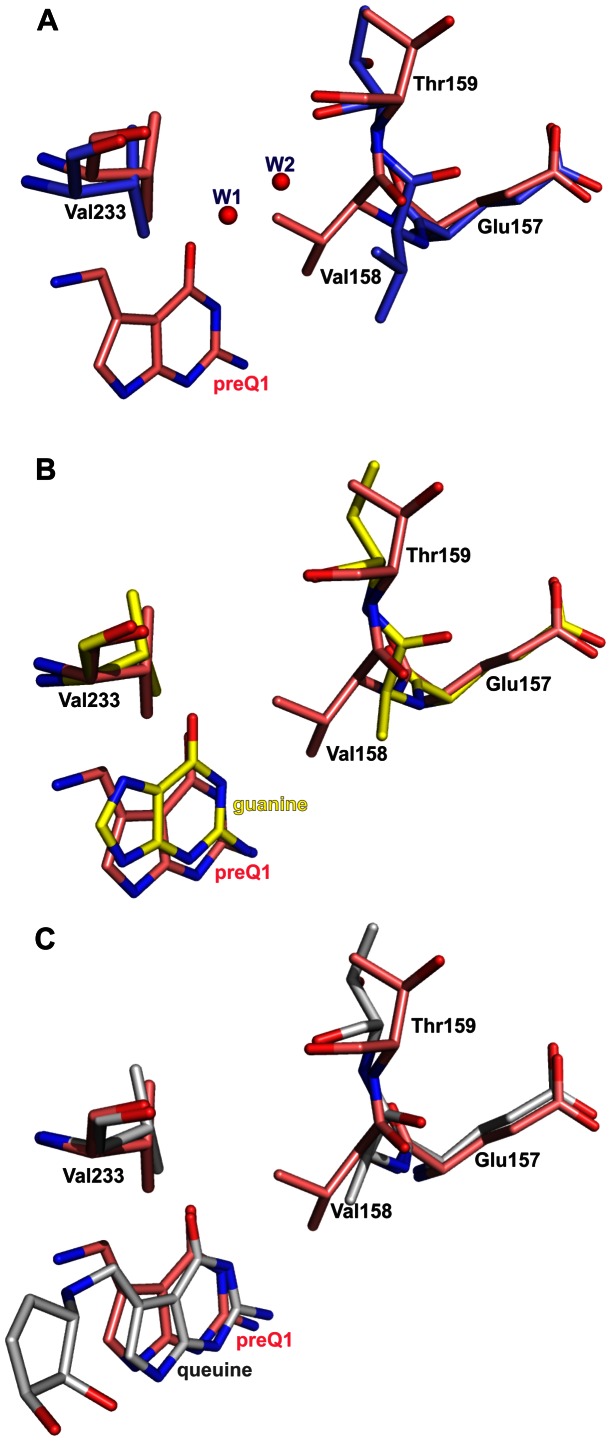
Superimposition of preQ_1_-bound Tgt(Cys158Val) with its apo-, its guanine- and its queuine-bound form. Carbon atoms of the Tgt(Cys158Val)·preQ_1_ complex are coloured pink in all sub-figures. (A) Superimposition (based on c_α_) of preQ_1_-bound Tgt(Cys158Val) and apo-Tgt(Cys158Val). Carbon atoms of apo-Tgt(Cys158Val) are coloured blue. Binding of preQ_1_ to Tgt(Cys158Val) provokes a shift of Val158 towards the ligand leading to the displacement of two water molecules (W1 and W2; shown as red spheres) which are present within this region in apo-Tgt(Cys158Val) and absent in the complex structure. In addition, the side chain of the proximate Thr159 rotates by about 90°. Exactly the same structural changes upon binding of preQ_1_ are observed for Tgt(Cys158Val/Val233Gly). (B) Superimposition (based on c_α_) of preQ_1_-bound Tgt(Cys158Val) and guanine-bound Tgt(Cys158Val). Carbon atoms of the Tgt(Cys158Val)·guanine complex are coloured yellow. In the Tgt(Cys158Val)·guanine complex, the side chain of Val158 becomes largely disordered. The Thr159 side chain adopts a similar conformation as observed in the apo-structure. (C) Superimposition (based on c_α_) of preQ_1_-bound Tgt(Cys158Val) and queuine-bound Tgt(Cys158Val). Carbon atoms of the Tgt(Cys158Val)·queuine complex are shown in grey. Binding of queuine obviously leads to disordering of the Val158 side chain as no electron density attributable to this isopropyl moiety is present in the electron density map of the refined Tgt(Cys158Val)·queuine complex structure. Also upon binding of queuine no conformational change of the Thr159 side chain is observed. It adopts a similar conformation as in the apo- and guanine-bound structures.

A conspicuous structural change upon binding of preQ_1_ is observed within the binding pockets of those Tgt variants which harbour the Cys158Val mutation. Both in Tgt(Cys158Val) and Tgt(Cys158Val/Val233Gly) the binding of preQ_1_ provokes a shift of Val158 towards the ligand enabling five Van-der-Waals interactions of its isopropyl side chain with the 7-deazaguanine scaffold of preQ_1_ (3.5 Å to 4.3 Å calculated by CONTACSYM [41]). In addition, a noticeable change of the proximate Thr159 whose side chain is rotated by about 90° is observed (Figure 9A). So far, in no other crystal structure of *Z. mobilis* Tgt or any variant thereof such a conformational change of Thr159 has been observed upon ligand binding. To obtain indication of whether the reduced affinity of Tgt(Cys158Val) and Tgt(Cys158Val/Val233Gly) towards preQ_1_ arises from the described structural changes, we determined the crystal structure of Tgt(Cys158Val) in complex with guanine. If the observed conformational rearrangements actually account for the decrease in affinity to preQ_1_ we expected them to not occur upon guanine binding since the Cys158Val exchange has no measurable impact on guanine recognition. The crystal structure of the Tgt(Cys158Val)·guanine complex reveals that upon guanine binding the side chain of Val158 becomes largely disordered as it is ill-defined in the electron density map. The position of the main chain atoms of Val158 indicates that there is hardly any shift of this residue towards the ligand. Strikingly, no change of the Thr159 side chain conformation which closely resembles that of the apo-structure is observed (Figure 9B). We would like to note that binding of guanine in Tgt(Cys158Val), as in wild type Tgt, leads to a Leu231/Ala232 peptide bond conformation which is flipped compared to the preQ_1_-bound situation [22]. In the preQ_1_-bound enzyme the carbonyl group of this peptide bond is exposed to the binding pocket while the backward-oriented amide forms an *H* bond to the deprotonated side chain carboxyl of Glu235. In contrast, upon guanine binding the *NH*-group of this peptide bond is exposed, while the backward-oriented carbonyl oxygen interacts with the protonated Glu235 side chain carboxyl group (see also Figure 3).

Ultimately, we determined the crystal structures of the studied Tgt variants in complex with queuine. Although electron density which can be attributed to a bound ligand is clearly present in the binding pocket of the refined structure of “wild type” Tgt crystallised in the presence of queuine it turned out to be too ill-defined to allow a definite placement of queuine in the model (Figures 7B and 8B). In contrast, the electron density map of the Tgt(Cys158Val)·queuine complex structure allowed the placement of the 7-deazaguanine scaffold and the exocyclic aminomethyl group of queuine. Clearly, this part of the ligand shows, as expected, a binding mode which strongly resembles that of preQ_1_ (Figures 7D and 8D). Only rudimentary density, however, is visible for the dihydroxy-cyclopentenyl moiety of queuine probably due to its enhanced residual mobility. Yet, the position of the aminomethyl group implicates the dihydroxy-cyclopentenyl pointing towards the uracil 33 nucleotide subpocket of the enzyme. This leads, compared to the preQ_1_-bound situation, to the displacement of a water molecule which forms an *H* bond to the exocyclic amino group of preQ_1_. This water is strictly conserved in the preQ_1_ complex structures of all investigated Tgt variants and designated “W” in Figures 8A, 8C, 8E and 8G. Superimposition of the queuine-bound Tgt(Cys158Val) structure with the structure of Tgt in complex with RNA [21] reveals that in the present conformation the dihydroxy-cyclopentenyl moiety of queuine sterically interferes with the ribose of the uracil 33 nucleotide and the phosphate at position 34. Hence, the binding of queuine in the observed conformation is obviously not compatible with the binding of a tRNA substrate. Notably, as observed for guanine, the presence of queuine in the substrate pocket of Tgt(Cys158Val) apparently completely disorders the Val158 side chain as there is no electron density assignable to this isopropyl moiety (Figure 8D). In contrast, this side chain is excellently defined in the electron density maps of apo- and preQ_1_-bound Tgt(Cys158Val) and Tgt(Cys158Val/Val233Gly). The positioning of the Val158 main chain atoms, however, suggests a significant shift of this residue towards the bound ligand although less pronounced as observed in the preQ_1_ complex structure of Tgt(Cys158Val). In accordance with the structure of the guanine-bound Tgt(Cys158Val), this shift is not concomitant with a rotation of the Thr159 side chain (Figure 9C).

Also the electron density maps of the Tgt(Val233Gly)·queuine and Tgt(Cys158Val/Val233Gly)·queuine complex structures enabled reliable placement of the 7-deazaguanine scaffold of the ligand which is, in both cases, similarly bound as in the Tgt(Cys158Val)·queuine complex. In both maps fragmentary density is visible for the dihydroxy-cyclopentenyl substituent (Figures 7F, 7H as well as 8F, 8H). Figures 7F and 7H show that the mutation of Val233 to glycine results in a considerable gain of space in the substrate binding pocket which allows the dihydroxy-cyclopentenyl of queuine to occupy the cavity located between Gly233 and the side chain of Cys/Val158. Within this cavity the dihydroxy-cyclopentenyl apparently has no specific interaction with the protein. As a consequence, it seems to be highly flexible which is reflected by the ill-defined electron density assigned to this moiety in both structures. As in the structure of queuine-bound Tgt(Cys158Val), nearly no electron density can be attributed to the side chain of Val158 in the Tgt(Cys158Val/Val233Gly)·queuine complex structure, indicating an increased mobility of this residue upon the binding of queuine. Also, in agreement with the structure of queuine-bound Tgt(Cys158Val), the positioning of the Val158 main chain atoms suggests a shift of this residue towards the ligand which is not concomitant with a rotation of the Thr159 side chain.

It must be noted that in the case of all queuine complex structures determined in this study the electron density assigned to the 7-deazaguanine scaffold of the ligand by far does not comply with the nominal resolution of the respective structure. Even in the case of the mutated variants where reliable placement of the scaffold into the binding pocket was possible the electron density within this area appears rather fuzzy or even patchy and lacks detail. It is likely that multiple conformations and the low occupancy (on average 60%) of queuine in the binding pocket provoked the poor electron density.

## Discussion

While eucaryotic Tgt exchanges the genetically encoded guanine by queuine at position 34 of tRNAs^His,Tyr,Asp,Asn^, bacterial Tgt is not able to insert the latter hypermodified base into these tRNAs. Instead, it uses the queuine-precursor preQ_1_ as a substrate which is then further converted to the final queuine at the tRNA level. A homology model of the *C. elegans* Tgt catalytic subunit suggests that the exchange of Cys158 and Val233 in bacterial Tgt (*Z. mobilis* Tgt numbering) by valine and glycine, respectively, in eucaryotic Tgt largely accounts for the difference in substrate specificity between the Tgt enzymes of both domains of life [35]. This assumption was supported by a homology model of the human Tgt catalytic subunit created in the present study. The replacement of Val233 by glycine results in a considerable gain of space in the substrate binding pocket most probably required for the accommodation of the dihydroxy-cyclopentenyl moiety of queuine. The side chain of the valine residue corresponding to Cys158 of *Z. mobilis* Tgt appears at a position where it might form an interaction with the dihydroxy-cyclopentenyl substituent. While Cys158 and Val233 are highly conserved in bacterial Tgt, the corresponding valine and glycine residues are invariant in all eucaryotic Tgt catalytic subunits whose sequences have been determined so far [42].

Based on the homology models of the *C. elegans* and human Tgt catalytic subunits, we replaced, *via* site-directed mutagenesis, Val233 and Cys158 in *Z. mobilis* Tgt by glycine and valine, respectively. In addition, to separately investigate the influence of these amino acid exchanges on substrate specificity and catalysis, we created two further variants, each containing solely one of the two mutations. However, the introduced changes which we desired for to convert substrate selectivity of bacterial towards eucaryotic Tgt did not result in an enzyme that is able to insert queuine into tRNA. Instead, the amino acid exchanges mainly interfere with catalysis by reducing *k*_cat_(guanine) and *k*_cat_(preQ_1_). While the reduction is not of great significance for Tgt(Cys158Val) (2- to 3-fold), it accounts for one order of magnitude for Tgt(Val233Gly) and for two for Tgt(Cys158Val/Val233Gly). The crystal structures of these Tgt variants, determined in complex with preQ_1_, clearly show that preQ_1_ is, in each case, identically positioned as in the wild type, ruling out a substrate misorientation with respect to any catalytically relevant residue. Instead, the mutated residues may exert influence on the properties of a nearby catalytic water molecule acting as a proton donor during guanine excision (Figures 3A and 3B).

A notable consequence of the Cys158Val exchange is the significantly decreased affinity of the enzyme towards preQ_1_ but not guanine. A similar phenomenon was reported by Chen *et al.* [26] who mutated the corresponding cysteine of *E. coli* Tgt to valine, resulting in a dramatical loss of affinity to preQ_1_. Consistently, a measurable gain in preQ_1_ affinity was observed by this group for the human enzyme upon mutation of the corresponding Val161 to cysteine. The study does, however, not provide any data demonstrating the impact of these mutations on guanine as a substrate. As already proposed by Chen *et al.* [26], it seems likely that, in eucaryotes, a valine at the mentioned position in the catalytic subunit of eucaryotic Tgt preferably prevents the (irreversible!) insertion of preQ_1_ into tRNA. Our data provide evidence that this is achieved without the loss of affinity towards guanine which, most likely, would result in an attenuated affinity to the unmodified substrate tRNAs comprising guanine at position 34.

The crystal structures of Tgt(Cys158Val) and Tgt(Cys158Val/Val233Gly), each in complex with preQ_1_, reveal, compared to the corresponding apo-forms, a shift of Val158 towards the ligand. This allows the isopropyl side chain of this residue to form Van-der-Waals contacts to the substrate. This structural change is concomitant with a ca. 90° rotation of the Thr159 side chain thus resulting in a novel conformation which has, until now, not been observed in any other crystal structure of *Z. mobilis* Tgt or a mutated variant thereof. The crystal structure of Tgt(Cys158Val) in complex with guanine reveals that there is no conformational change of Thr159 upon guanine binding. Obviously, the Thr159 conformational change is triggered by preQ_1_ and not by guanine. This observation is of greater remarkability as the Thr159 side chain remains at large distance without any direct contact to the aminomethyl group of preQ_1_. The *Penultimate Rotamer Library* published by Lovell *et al.* [43] reports three preferred rotamers for threonine observed with a relative frequency of 7%, 43%, and 49%. Notably, in the preQ_1_ complexes of Tgt(Cys158Val) and Tgt(Cys158Val/Val233Gly) Thr159 adopts the least populated conformer, which probably corresponds to the rotamer that is least energetically favourable. In contrast, the Thr159 side chain conformation observed in all other crystal structures of *Z. mobilis* Tgt matches with the rotamer found with a frequency of 43%. Accordingly, the side chain conformation of Thr159 induced upon preQ_1_ binding may well be correlated with the reduced preQ_1_ affinity observed upon mutation of Cys158 to valine. It should be noted, that Thr159 is in most eucaryotic Tgt catalytic subunits replaced by a nearly isosteric valine residue [42].

As neither *Z. mobilis* Tgt nor any of our mutated variants are able to use queuine as a substrate we investigated whether or not queuine is actually recognised by these enzymes. For that purpose, we tested if queuine was able to act as inhibitor of bacterial Tgt since its binding to the active site should inevitably prevent radiolabelled guanine from being inserted into tRNA. Indeed, a considerable inhibitory effect of queuine on Tgt activity was observed for all investigated enzyme variants including “wild type” Tgt. The recognition of queuine in the substrate binding pocket of “wild type” Tgt and Tgt(Cys158Val) seems surprising as these variants do not exhibit the space-opening Val233Gly exchange believed to be prerequisite for the accommodation of the dihydroxy-cyclopentenyl side chain of queuine. Trapping experiments revealed that queuine predominantly acts as a competitive inhibitor with respect to tRNA as a substrate on all studied Tgt variants. This means queuine binds to the respective apo-enzymes but hardly to the covalent enzyme·tRNA complexes formed upon the release of guanine. Solely in the case of Tgt(Cys158Val/Val233Gly), the variant that should best approach the eucaryotic enzyme, the result of the trapping experiment indicates that the covalent enzyme·tRNA complex may, though to a low extent, be able to bind queuine.

The crystal structure of Tgt(Cys158Val) in complex with queuine provides an evident explanation why this variant is able to bind queuine exclusively in its apo-form. The bulky side chain of Val233 does not prevent queuine from binding to the active site. Yet, it forces its dihydroxy-cyclopentenyl moiety into a position which is not compatible with tRNA binding due to clashes with the tRNA sugar phosphate backbone. The same is likely to be true for “wild type” Tgt although ill-defined electron density in this crystal structure does not allow a reliable placement of queuine into the substrate binding pocket of the refined model.

The crystal structures of Tgt(Val233Gly) and Tgt(Cys158Val/Val233Gly) with queuine reveal that the Val233Gly exchange allows the dihydroxy-cyclopentenyl substituent to occupy the created cavity between position 233 and 158. The adopted binding mode of queuine will not interfere sterically with the sugar-phosphate backbone of bound tRNA. Yet, the results of the trapping experiment suggest that Tgt(Val233Gly) is also not able to bind queuine after the formation of the covalent enzyme·tRNA complex. Tgt(Cys158Val/Val233Gly) is, if at all, only to a minor extent able to bind queuine after the formation of this complex. The fuzzy electron density corresponding to the bound queuine suggests high residual ligand flexibility accompanied by a significant disorder induced in the active sites of the studied Tgt variants. The latter becomes evident from elevated *B*-factors and ill-defined electron density of residues lining the substrate binding pocket. Possibly, the enhanced mobility of the substrate binding pocket and of the bound queuine in these complexes is not compatible with the constrictions induced by the formation of the covalent enzyme·tRNA complex. Anyway, the fact that queuine is not able to bind efficiently to the covalent complex of any Tgt variant provides a plausible explanation why it does not constitute a substrate. None of the crystal structures determined in this study nor the *in silico* model of the catalytic subunit of human Tgt provide any clue on how this problem is overcome by eucaryotic Tgt. Probably, further residues, that are more distant from the active centre but involved in positioning the tRNA substrate have to be changed as well to enable bacterial Tgt to insert queuine into tRNA. In addition, it has to be pointed out once more that eucaryotic Tgt not only consists of a catalytic subunit, which is closely related to the bacterial protomer but, in addition, also contains a second subunit, which is catalytically inactive. This second subunit might possibly be prerequisite to enable the enzyme to use queuine as a substrate by aligning the tRNA substrate in a manner that somehow compensates for the loss of activity observed in our mutated variants.

Comparison of the active centre of *Z. mobilis* Tgt with the modelled human Tgt catalytic subunit reveals that Ala232 adjacent to Val233 in the bacterial enzyme is replaced by a serine (Ser231) in human Tgt (Figure 4). This residue is involved in a peptide switch necessary to accommodate preQ_1_ after the excised guanine 34 left the binding pocket [22] (Figure 3). Although the main chain amide of Ala232 binds indirectly, *via* a water molecule, to *N*7 of guanine 34 its side chain methyl group forms no interaction with any substrate. As this residue is replaced by a serine in many bacterial Tgts too, it seems unlikely that it contributes to substrate specificity or catalytic activity. Nevertheless, we introduced the Ala232Ser mutation into Tgt(Cys158Val/Val233Gly) to investigate its impact. For comparison, the Ala232Ser mutation was also introduced into “wild type” Tgt. Kinetic measurements showed that the Ala232Ser conversion further reduced the catalytic activity of Tgt(Cys158Val/Ala232Ser/Val233Gly). The residual turnover observed for this variant with both guanine and preQ_1_ as substrates was too small to permit the determination of reliable Michaelis-Menten parameters, due to the low signal to noise ratio of the measured data. Interestingly, a pronounced 10^2^-fold decrease in *k*_cat_ compared to “wild type” Tgt was also observed for Tgt(Ala232Ser) using guanine or preQ_1_ as a substrate (data not shown). Therefore, the Ala232Ser variants of Tgt were not considered further in this study. In addition, crystal structures that were determined of Tgt(Cys158Val/Ala232Ser/Val233Gly) in its apo-form, in complex with preQ_1_ and in complex with queuine do not show any significant differences to the corresponding ones of Tgt(Cys158Val/Val233Gly) and, due to redundance, will not be discussed in detail (data statistics and PDB codes of these structures are provided in Table S2 of the Supporting Information). Nevertheless, it is worth mentioning that, in the Tgt(Cys158Val/Ala232Ser/Val233Gly)·preQ_1_ complex the above-mentioned side chain rotation of Thr159, thought to correlate with the Cys158Val mutation upon binding of preQ_1_, is also observed. This conformational change is not visible in the structure of this variant in complex with queuine.

## Materials and Methods

### Homology modelling

The crystal structure of *Z. mobilis* Tgt (PDB code **1r5y** chain A) was used as a template to create a model of the human Tgt catalytic subunit (UniProtKB/Swiss-Prot [44] accession code Q9BXR0). Using ClustalW 1.83 [45] the corresponding sequences aligned with 42% identity. Based on the alignment, ten homology models for human Tgt were calculated with MODELLER 6a [46]. As no significant differences were apparent, the model with the lowest MODELLER target function value (11,429.19) was selected as representative. The coordinates of the homology model are provided within the Supporting Information (Coordinates S1).

### Cloning and Tgt preparation

Site-directed mutagenesis was performed using the QuickChange™ kit (Stratagene) following the vendor's protocol. The oligonucleotides listed in Table 5 were used to introduce the mutations into the *Z. mobilis tgt*(Tyr106Phe) expression plasmid pET9d-ZM-Y106F [37]. Sequence analysis of the entire *tgt* gene (MWG Biotech, Ebersberg) confirmed in each case the presence of the desired mutation(s) as well as the absence of any further unwanted mutation. Subsequently, the mutated plasmids were transformed into *E. coli* BL21(DE3)/pLysS cells. These cells were used for the preparation of the mutated Tgt variants following the protocol of Romier *et al.* [47].

**Table 5:** Oligonucleotides used in mutagenesis.

mutation/name of oligo	Sequence
C158V-f	5′-GTA ATG GCC TTT GAC GAA GTC ACG CCT TAT CCA GC-3′
C158V-b	5′-GC TGG ATA AGG CGT GAC TTC GTC AAA GGC CAT TAC-3′
V233G-f	5′-GCT GTT GGG GGA TTG GCT GGG GGT GAA GGA CAG GAT G-3′
V233G-b	5′-C ATC CTG TCC TTC ACC CCC AGC CAA TCC CCC AAC AGC-3′
A232S-f	5′-GCT GTT GGG GGA TTG TCT GTG GGT GAA GGA CAG GAT G-3′
A232S-b	5′-C ATC CTG TCC TTC ACC CAC AGA CAA TCC CCC AAC AGC-3′
A232S-V233G-f	5′-GCT GTT GGG GGA TTG TCT GGG GGT GAA GGA CAG GAT G-3′
A232S-V233G-b	5′-C ATC CTG TCC TTC ACC CCC AGA CAA TCC CCC AAC AGC-3′

Bases deviating from the original *tgt* sequence are underlined.

### Preparation of *Escherichia coli* tRNA^Tyr^

Preparation of unmodified *E. coli* tRNA^Tyr^ (ECY2) [48] *via in vitro* transcription was done using the RiboMAX™ Large Scale RNA Production System-T7 (Promega) according to the vendor's protocol. The concentration of tRNA was determinded *via* UV photometry (λ = 280 nm).

### Preparation of preQ_1_ and queuine

Preparation of preQ_1_ and queuine was carried out according to [38].

### Determination of kinetic parameters

Progress curves of Tgt activity using guanine and tRNA^Tyr^ as substrates were measured by monitoring incorporation of radiolabelled guanine into tRNA^Tyr^. The reaction proceeded at 37°C in 75 µL solutions containing 150 nmol·L^−1^ (of monomer) *Z. mobilis* Tgt [300 nmol·L^−1^ in case of Tgt(Cys158Val/Val233Gly)] and variable concentrations of *E. coli* tRNA^Tyr^ and guanine (7.5% [8-^3^ *H*]-guanine; 12 Ci/mmol, Hartmann Analytic). The assay was performed in 200 mmol·L^−1^ HEPES buffer pH 7.3, 20 mmol·L^−1^ MgCl_2_, and 0.037% (v/v) Tween 20 (Roth). Reactions were started by adding tRNA and [8-^3^ *H*]-guanine to the protein solution. To allow the protein solution to adjust to assay temperature it was pre-incubated at 37°C for 10 min prior to substrate addition. After the reactions were started, 15 µL aliquots were taken at intervals of 1 to 4 min. Aliquots were immediately transferred to Whatman GC-F glass microfiber filters (GE Healthcare) and quenched with 10% (w/v) trichloroacetic acid at 0°C for 15 min. Unbound guanine was washed from the filters in 7 min intervals twice with 5% (w/v) trichloroacetic acid and twice with technical grade ethanol. Filters were dried at 60°C for 45 min. ^3^ *H* incorporated into tRNA was quantified using liquid scintillation counting. Results from the aliquots were used to calculate initial velocity using GraFit (V. 4.09; Erithacus Software Ltd., 1999).

Michaelis-Menten parameters for tRNA and guanine were determined separately in duplicate and average values were calculated. Kinetic parameters for guanine were measured using 15 µmol·L^−1^ tRNA^Tyr^ and concentrations of [8-^3^ *H*]-guanine varying in the range of 0.5–20 µmol·L^−1^. Kinetic parameters for tRNA were measured using 20 µmol·L^−1^ [8-^3^ *H*]-guanine and tRNA^Tyr^ concentrations varying in the range of 0.25–15 µmol·L^−1^. Initial velocities in “counts per min” were transferred to µmol·L^−1^·min^−1^ using a calibration constant derived from liquid scintillation counting of [8-^3^ *H*]-guanine solutions with variable concentrations. Kinetic parameters were determined *via* double-reciprocal linearisation and linear regression using GraFit.

Michaelis-Menten parameters for preQ_1_ were determined in the same manner as for guanine but using radiolabelled preQ_1_ ([methylene-^3^ *H*]-7-aminomethyl-7-deazaguanine = [^3^ *H*]-preQ_1_; 7.0 Ci/mmol, Moravek Biochemicals Inc., California, USA) as substrate base. The concentration of preQ_1_ was varied between 0.5–15 µmol·L^−1^ for “wild type” Tgt and Tgt(Val233Gly) or rather 1–50 µmol·L^−1^ for Tgt(Cys158Val) and Tgt(Cys158Val/Val233Gly).

In the same manner, enzymatic activity was analysed using [^3^ *H*]-queuine (7.0 Ci/mmol, Moravek Biochemicals Inc., California, USA) as substrate base at concentrations of up to 50 µmol·L^−1^. Alternatively, enzymatic activity with queuine as substrate base was measured *via* monitoring the removal of [8-^3^ *H*]-guanine from tRNA^Tyr^ radiolabelled in position 34 as described in [22] but with the substrate base preQ_1_ replaced by queuine. Radiolabelled tRNA^Tyr^ was used in a concentration of 8 µmol·L^−1^. The concentration of queuine amounted to 50 µmol·L^−1^ for all inspected Tgt variants.

### Determination of inhibition constants

Inhibition constants of queuine (competitive against tRNA) were determined for “wild type” Tgt and mutated variants as described in [23].

### SDS-PAGE trapping experiment

5 µmol·L^−1^ *Z. mobilis* Tgt (monomer), 100 µmol·L^−1^ *E. coli* tRNA^Tyr^ (ECY2) and, if necessary, 1 mmol·L^−1^ of queuine or rather 2,6-diamino-3*H*-quinazolin-4-one in 10 µL of 100 mmol·L^−1^ HEPES buffer, pH 7.3, 20 mmol·L^−1^ MgCl_2_, and 5 mmol·L^−1^ dithiothreitol were incubated for 1 h at 25°C. A total of 10 µL of SDS loading buffer [250 mmol·L^−1^ TrisHCl pH 6.8, 8% (w/v) SDS, 40% (v/v) Glycerol, 0.04% (w/v) bromophenol blue, 8% (v/v) 2-sulfanylethanol] was added and incubated for another hour at 25°C to allow proper unfolding. 10 µL of each sample were then loaded onto a 15% SDS polyacrylamide gel and electrophoresis was performed with SDS running buffer [25 mmol·L^−1^ TrisHCl pH 8.8, 200 mmol·L^−1^ glycine, 0.1% (w/v) SDS]. Gels were stained with Coomassie® brilliant blue R-250 (Bio-RAD).

### Microscale thermophoresis measurements

*Z. mobilis* Tgt at a concentration of 10 µmol·L^−1^ was labelled with Alexa Fluor®647 succinimidyl ester at a concentration of 40 mg·L^−1^ at room temperature for 30 min in a 500 mmol·L^−1^ NaCl solution buffered with 50 mmol·L^−1^ HEPES pH 8.1 (molar dye ∶ protein ratio≈3 ∶ 1). Excessive unreacted Alexa Fluor®647 was removed with a NAP5 sephadex column (GE Healthcare) equilibrated with 2 mol·L^−1^ NaCl, 1 mmol·L^−1^ EDTA solution buffered with 10 mmol·L^−1^ TrisHCl pH 7.8. Photometry at 650 nm and 280 nm indicated a label ∶ protein ratio of 0.8. The Alexa Fluor®647-Tgt solution was adjusted to 100 nmol·L^−1^ with 100 mmol·L^−1^ HEPES pH 7.3 buffer containing 20 mmol·L^−1^ MgCl_2_ and 0.037% (v/v) (≡323 µmol·L^−1^) Tween 20 (Roth). The final solution contained NaCl at a concentration of approximately 300 mmol·L^−1^. The tRNA^Tyr^ ligand was dissolved in the same buffer (free of NaCl) at a concentration of 10 µmol·L^−1^. A series of fifteen 1 ∶ 1 dilutions of tRNA^Tyr^ solution ∶ buffer solution was prepared producing tRNA^Tyr^ concentrations ranging from 305 pmol·L^−1^ to 10 µmol·L^−1^. For thermophoresis, each of these solutions was mixed with one volume of Alexa Fluor®647-Tgt solution resulting in a constant concentration of fluorescence labelled Tgt of 50 nmol·L^−1^ and tRNA^Tyr^ concentrations ranging from 153 pmol·L^−1^ to 5 µmol·L^−1^. After 10 min incubation followed by centrifugation at 10 000× g for 10 min, approximately 2 µL of each solution were filled into Monolith NT Standard treated capillaries (NanoTemper Technologies GmbH). Thermophoresis (including temperature jump) was measured at room temperature for 10 s by means of a Monolith NT.015 instrument (NanoTemper Technologies GmbH), using 100% LED power and 60% infrared laser power. Data of three independent runs were averaged and analysed using Origin 7 (Origin Lab). Curve fitting and *K*_D_ calculation was done using the program NanoTemper Analysis 1.2.009 (NanoTemper Technologies GmbH) based on the following equation [49]:

*T* = thermophoresis signal

*U* = minimal signal (unbound protein)

*B* = maximal signal (protein saturated with ligand)

*c*_p_ = concentration of labelled protein

*c*_i_ = concentration of ligand

*K*_D_ = dissociation constant
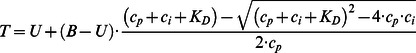



### Crystallisation, data collection, and processing

Crystals of the investigated Tgt variants suitable for ligand soaking were grown *via* the hanging-drop vapour diffusion method. Droplets were prepared by mixing 1 µL of concentrated protein solution (12 to 15 mg·mL^−1^ in 2 mol·L^−1^ NaCl, 10 mmol·L^−1^ Tris/HCl pH 7.8, 1 mmol·L^−1^ EDTA, 1 mmol·L^−1^ DTT) with 1 µL reservoir solution (11% (w/v) PEG 8,000, 100 mmol·L^−1^ Tris/HCl, pH 8.5, 1 mmol·L^−1^ DTT, 10% (v/v) DMSO). Crystals grew within one week at 18°C in the presence of 1.0 mL of reservoir solution.

For cocrystallisation of Tgt(Cys158Val) with guanine, guanine·HCl was dissolved in DMSO and added to the crystallisation droplet to a final concentration of 5 mmol·L^−1^. As guanine is, under the above-named conditions, not soluble up to this concentration a substantial portion of the compound precipitated. Crystals grew within the guanine precipitate in two weeks. To allow cocrystallisation of the Tgt variants with Q the compound was dissolved in DMSO and added to the droplet to a final concentration of 20 mmol·L^−1^. Crystals of the Tgt variants complexed to preQ_1_ were produced by a soaking procedure. The compound was dissolved in DMSO and added to 2 µL of reservoir solution to a final concentration of 10 to 20 mmol·L^−1^. Finally, a single pre-grown Tgt crystal was transferred into the droplet which was sealed against 1.0 mL of reservoir solution and soaked for 30 min.

For data collection, crystals were cryoprotected using glycerol. The glycerol and PEG 8,000 concentrations of the reservoir buffers were increased stepwise by transferring the crystals to six different 2 µL cryodroplets each with 30-min incubation times while sealed against 1.0 mL of the same solution (glycerol concentrations (v/v): 5%→10%→15%→20%→25%→30%; and PEG 8,000 concentrations (w/v): 5.0%→6.3%→7.5%→8.0%→8.8%→9.8%, respectively). These droplets also contained the ligands at the same concentrations as the soaking and cocrystallisation solutions. The cryo-soaked crystals were flash-frozen in liquid nitrogen. Data sets (**3bld**, **4dg0**, **4e2v**, **4hvx**, **4h7z**, **4h6e**, **4hsh**, **4hqv**, **4gcx** and “WT”-Tgt·queuine) were collected at cryo conditions (−173°C≡100 K) at the BESSY II (Helmholz-Zentrum, Berlin, Germany) beamlines BL-14.1 (λ = 0.91841 Å) and BL-14.2 (λ = 0.91841 Å) and with CuKα radiation (λ = 1.5418 Å) using a Rigaku RU-300 rotating-anode generator at 50 kV and 90 mA equipped with Xenocs focussing optics and an R-AXIS IV detector. All crystals tested exhibited monoclinic symmetry in space group *C*2 containing one monomer per asymmetric unit with Matthews coefficients of 2.3–2.4. Data processing and scaling was performed using the HKL2000 package [50] except for structure **4h7z** which was processed using iMOSFLM 1.0.6 [51] and SCALA [52]. For all refined structures unit cell dimensions for the crystals, data collection and processing statistics are given in Tables 2, 3 and 4.

Coordinates of the apo-Tgt crystal structure (PDB-code: **1pud** in case of the structures **3bl3**, **3bld**, **3blo**) or rather coordinates of Tgt in complex with 2-[(thiophen-2-ylmethyl)amino]-1,7-dihydro-8H-imidazo[4,5-g]quinazolin-8-one (PDB-code: **3gev** in case of structures **4dg0**, **4e2v**, **4hvx**, **4h7z**, **4h6e**, **4hsh**, **4hqv**, **4gcx** and “WT”-Tgt·queuine) were slightly modified (deletion of water and ligand as well as coordinates at the sites of mutation) before applying for initial rigid-body refinement of the protein molecule followed by repeated cycles of conjugate gradient energy minimisation, simulated annealing and *B*-factor refinement using the CNS program package [53]. Refinement at the later stages was performed with SHELXL [54] for structures **3bl3**, **3bld**, and **3blo**. Here, up to 50 cycles of conjugate gradient minimisation were performed with default restraints on bonding geometry and *B*-values: 5% of all data were used for *R*_free_ calculation. For the structures **4e2v**, **4dg0**, **4h7z**, **4h6e**, **4hqv**, **4hsh**, **4hqv**, **4gcx** and “WT”-Tgt·queuine refinement was performed with PHENIX 1.8-1069 [55] with 5% to 10% of all data being used for *R*_free_ calculation and followed by repeated cycles of maximum likehood energy minimization. For the structures **4h7z**, **4hvx**, **4hqv**, **4hsh** an additional optimization of the weights between X-ray target and stereochemistry as well as between ADP restraints was performed by PHENIX 1.8-1069 [55]. Amino acid side chains were fitted to σA-weighted 2|F_o_| - |F_c_| and |F_o_| - |F_c_| electron density maps using Coot [56]. Ligands were generated using SYBYL 8.0 (Tripos International: 1699 South Hanley Rd., St. Louis, Missouri, 63144, USA). Afterwards, geometric restraints of the ligands were calculated by Monomer Library Sketcher (ccp4) [57]. For structures **4e2v**, **4dg0**, **4h7z**, **4h6e**, **4hqv**, **4hsh**, **4hqv**, **4gcx** and “WT”-Tgt·queuine water was located using the refinement settings “update waters” implemented in the program PHENIX 1.8-1068 [55]. After further refinement cycles water was checked visually. Glycerol molecules and ligands were located in the difference electron density and added to the model for further refinement cycles. The temperature factors (individual *B*-factors) for structures **4e2v**, **4gcx**, **4gd0** and **3bld** were anisotropically refined, whereas for structures **4hvx**, **4h7z**, **4hsh**, **4hqv** and **4h6e** and “WT”-Tgt·queuine TLS refinement was applied. The definition of the TLS groups was done with the TLSMD server [58]. Restraints were applied to bond lengths and angles, planarity of aromatic rings, and Van-der-Waals contacts. Multiple side-chain conformations were built in case an appropriate electron density was observed and maintained during refinement and if the minor populated side chain showed at least 20% occupancy. All final models were validated using PROCHECK [59]. Analysis of temperature factors was done with Moleman [60].

### Alignment and Figures

Alignment of structures based on c_α_ with similar or identical sequences was performed with the alignment function implemented in Pymol (http://www.pymol.org). Figures were prepared using ChemDraw Std 12.0 (PerkinElmer, Massachusetts, USA) and Pymol.

### Protein Data Bank Accession Codes

The Protein Data Bank (PDB) accession codes allocated to the crystal structures determined in the course of this study are given in Tables 2, 3 and 4 and in Table S1.

## Supporting Information

Coordinates S1 **Coordinates of the homology model of the human Tgt catalytic subunit.**
(PDB)

Coordinates S2 **Coordinates of the** ***Z.mobilis*** **Tgt(Tyr106Phe) crystal structure determined in in the presence of queuine.**
(PDB)

Structure Factors S1 **Structure factors of the** ***Z.mobilis*** **Tgt(Tyr106Phe) crystal structure determined in in the presence of queuine.**
(MTZ)

Table S1 **Overview of crystal structures analysed by Biela** ***et al.*** **(2013).**
(DOCX)

Table S2 **Crystallographic data collection and refinement statistics of further structures related to the study of Biela** ***et al.*** **(2013).**
(DOCX)

## References

[1] Stengl B, Reuter K, Klebe G (2005) Mechanism and substrate specificity of tRNA-guanine transglycosylases (TGTs): tRNA-modifying enzymes from the three different kingdoms of life share a common catalytic mechanism. ChemBioChem 6: 1926–1939. 16206323 10.1002/cbic.200500063

[2] Oliva R, Tramontano A, Cavallo L (2007) Mg^2+^ binding and archaeosine modification stabilise the G15-C48 Levitt base pair in tRNAs. RNA 13: 1427–1436. 17652139 10.1261/rna.574407 PMC1950755

[3] Nakanishi S, Ueda T, Hori H, Yamazaki N, Okada N, et al (1994) A UGU sequence in the anticodon loop is a minimum requirement for recognition by *Escherichia coli* tRNA-guanine transglycosylase. J Biol Chem 269: 32221–32227. 7528209

[4] Curnow AW, Garcia GA (1995) tRNA-guanine transglycosylase from *Escherichia coli*. Minimal tRNA structure and sequence requirements for recognition. J Biol Chem 270: 17264–17267. 7615526 10.1074/jbc.270.29.17264

[5] Phillips G, Yacoubi BE, Lyons B, Alvarez S, Iwata-Reuyl D, et al (2008) Biosynthesis of 7-deazaguanosine-modified tRNA nucleosides: a new role for GTP cyclohydrolase I. J Bacteriol 190: 7876–7884. 18931107 10.1128/JB.00874-08 PMC2593212

[6] McCarthy RM, Somogyi A, Bandarian V (2009) *Escherichia coli* QueD is a 6-carboxy-5,6,7,8-tetrahydropterin synthase. Biochemistry 48: 2301–2303. 19231875 10.1021/bi9001437 PMC3227869

[7] McCarthy RM, Somogyi A, Lin G, Jacobsen NE, Bandarian V (2009) The deazapurine biosynthetic pathway revealed: *in vitro* enzymatic synthesis of preQ_0_ from guanosine 5′-triphosphate in four steps. Biochemistry 48: 3847–3852. 19354300 10.1021/bi900400e PMC2693876

[8] Van Lanen SG, Reader JS, Swairjo MA, de Crécy-Lagard V, Lee B, et al (2005) From cyclohydrolase to oxidoreductase: discovery of nitrile reductase activity in a common fold. Proc Natl Acad Sci USA 102: 4264–4269. 15767583 10.1073/pnas.0408056102 PMC555470

[9] Lee BWK, Van Lanen SG, Iwata-Reuyl D (2007) Mechanistic studies of *Bacillus subtilis* QueF, the nitrile oxidoreductase involved in queuosine biosynthesis. Biochemistry 46: 12844–12854. 17929836 10.1021/bi701265r

[10] Chikwana VM, Stec B, Lee BWK, de Crécy-Lagard V, Iwata-Reuyl D, et al (2012) Structural basis of biological nitrile reduction. J Biol Chem 287: 30560–30570. 22787148 10.1074/jbc.M112.388538 PMC3436371

[11] Van Lanen SG, Kinzie GS, Matthieu S, Link T, Culp J, et al (2003) tRNA modification by *S*-adenosylmethionine:tRNA ribosyltransferase-isomerase. Assay development and characterisation of the recombinant enzyme. J Biol Chem 278: 10491–10499. 12533518 10.1074/jbc.M207727200

[12] Mathews I, Schwarzenbacher R, McMullan D, Abdubek P, Ambing E, et al (2005) Crystal structure of *S*-adenosylmethionine:tRNA ribosyltransferase-isomerase (QueA) from *Thermotoga maritima* at 2.0 Å resolution reveals a new fold. Proteins 59: 869–874. 15822125 10.1002/prot.20419

[13] Grimm C, Ficner R, Sgraja T, Haebel P, Klebe G, et al (2006) Crystal structure of *Bacillus subtilis S*-adenosylmethionine:tRNA ribosyltransferase-isomerase. Biochem Biophys Res Comm 351: 695–701. 17083917 10.1016/j.bbrc.2006.10.096

[14] Frey B, McCloskey J, Kersten W, Kersten H (1988) New function of vitamin B12: cobamide-dependent reduction of epoxyqueuosine to queuosine in tRNAs of *Escherichia coli* and *Salmonella typhimurium* . J Bacteriol 170: 2078–2082. 3129401 10.1128/jb.170.5.2078-2082.1988 PMC211089

[15] Miles ZD, McCarthy RM, Molnar G, Bandarian V (2011) Discovery of epoxyqueuosine (oQ) reductase reveals parallels between halorespiration and tRNA modification. Proc Natl Acad Sci USA 108: 7368–7372. 21502530 10.1073/pnas.1018636108 PMC3088584

[16] Romier C, Reuter K, Suck D, Ficner R (1996) Crystal structure of tRNA-guanine transglycosylase: RNA modification by base exchange. EMBO J 15: 2850–2857. 8654383 PMC450223

[17] Ritschel T, Atmanene C, Reuter K, Van Dorsselaer A, Sanglier-Cianferani S, et al (2009) An integrative approach combining noncovalent mass spectrometry, enzyme kinetics and X-ray crystallography to decipher Tgt protein-protein and protein-RNA interaction. J Mol Biol 393: 833–847. 19627989 10.1016/j.jmb.2009.07.040

[18] Grädler U, Ficner R, Garcia GA, Stubbs MT, Klebe G, et al (1999) Mutagenesis and crystallographic studies of *Zymomonas mobilis* tRNA-guanine transglycosylase to elucidate the role of serine 103 for enzymatic activity. FEBS Lett 454: 142–146. 10413112 10.1016/s0014-5793(99)00793-0

[19] Grädler U, Gerber H-D, Goodenough-Lashua DAM, Garcia GA, Ficner R, et al (2001) A new target for Shigellosis: rational design and crystallographic studies of inhibitors of tRNA-guanine transglycosylase. J Mol Biol 306: 455–467. 11178905 10.1006/jmbi.2000.4256

[20] Goodenough-Lashua DM, Garcia GA (2003) tRNA-guanine transglycosylase from *E. coli*: a ping-pong kinetic mechanism is consistent with nucleophilic catalysis. Bioorg Chem 31: 331–344. 12877882 10.1016/S0045-2068(03)00069-5 PMC2784677

[21] Xie W, Liu X, Huang RH (2003) Chemical trapping and crystal structure analysis of a catalytic tRNA guanine transglycosylase covalent intermediate. Nature Struct Biol 10: 781–788. 12949492 10.1038/nsb976

[22] Tidten N, Stengl B, Heine A, Garcia GA, Klebe G, et al (2007) Glutamate versus glutamine exchange swaps substrate selectivity in tRNA-guanine transglycosylase: insight into the regulation of substrate selectivity by kinetic and crystallographic studies. J Mol Biol 374: 764–776. 17949745 10.1016/j.jmb.2007.09.062 PMC2100405

[23] Meyer EA, Donati N, Guillot M, Schweizer WB, Diederich F, et al (2006) Synthesis, biological evaluation, and crystallographic studies of extended guanine-based (*lin*-benzoguanine) inhibitors for tRNA-guanine transglycosylase (TGT). Helv Chim Acta 89: 573–597.

[24] Garcia GA, Chervin SM, Kittendorf JD (2009) Identification of the rate-limiting step of tRNA-guanine transglycosylase from *Escherichia coli* . Biochemistry 48: 11243–11251. 19874048 10.1021/bi901501a PMC2789984

[25] Okada N, Nishimura S (1979) Isolation and characterization of a guanine insertion enzyme, a specific tRNA transglycosylase, from *Escherichia coli* . J Biol Chem 254: 3061–3066. 107167

[26] Chen V-C, Brooks AF, Goodenough-Lashua DM, Kittendorf JD, Showwalter HD, et al (2011) Evolution of eucaryal tRNA-guanine transglycosylase: insight gained from the heterocyclic substrate recognition by the wild-type and mutant human and *Escherichia coli* tRNA-guanine transglycosylases. Nucleic Acids Res 39: 2834–2844. 21131277 10.1093/nar/gkq1188 PMC3074131

[27] Boland C, Hayes P, Santa-Maria I, Nishimura S, Kelly VP (2009) Queuosine formation in eucaryotic tRNA occurs *via* a mitochondria-localized heteromeric transglycosylase. J Biol Chem 284: 18218–18227. 19414587 10.1074/jbc.M109.002477 PMC2709355

[28] Chen V-C, Kelly VP, Stachura SV, Garcia GA (2010) Characterization of the human tRNA-guanine transglycosylase: confirmation of the heterodimeric subunit structure. RNA 16: 958–968. 20354154 10.1261/rna.1997610 PMC2856889

[29] Durand JM, Dagberg B, Uhlin BE, Björk GR (2000) Transfer RNA modification, temperature and DNA superhelicity have a common target in the regulatory network of the virulence of *Shigella flexneri*: the expression of the *virF* gene. Mol Microbiol 35: 924–935. 10692168 10.1046/j.1365-2958.2000.01767.x

[30] Hurt JK, Olgen S, Garcia GA (2007) Site-specific modification of *Shigella flexneri virF* mRNA by tRNA-guanine transglycosylase *in vitro* . Nucleic Acids Res 35: 4905–4913. 17626052 10.1093/nar/gkm473 PMC1950534

[31] Ritschel T, Hörtner S, Heine A, Diederich F, Klebe G (2009) Crystal structure analysis and *in silico* pKa calculations suggest strong pKa shifts of ligands as driving force for high-affinity binding to TGT. ChemBioChem 10: 716–727. 19199329 10.1002/cbic.200800782

[32] Ritschel T, Kohler PC, Neudert G, Heine A, Diederich F, et al (2009) How to replace the residual solvation shell of polar active site residues to achieve nanomolar inhibition of tRNA-guanine transglycosylase. ChemMedChem 4: 2012–2023. 19894214 10.1002/cmdc.200900343

[33] Kohler PC, Ritschel T, Schweizer WB, Klebe G, Diederich F (2009) High-affinity inhibitors of tRNA-guanine transglycosylase replacing the function of a structural water cluster. Chem Eur J 15: 10809–10817. 19746363 10.1002/chem.200901270

[34] Rakovich T, Boland C, Bernstein I, Chikwana VM, Iwata-Reuyl D, et al (2011) Queuosine deficiency in eukaryotes compromises tyrosine production through increased tetrahydrobiopterin oxidation. J Biol Chem 286: 19354–19363. 21487017 10.1074/jbc.M111.219576 PMC3103313

[35] Romier C, Meyer JE, Suck D (1997) Slight sequence variations of a common fold explain the substrate specificities of tRNA-guanine transglycosylases from the three kingdoms. FEBS Lett 416: 93–98. 9369241 10.1016/s0014-5793(97)01175-7

[36] Deshpande KL, Katze JR (2001) Characterization of cDNA encoding the human tRNA-guanine transglycosylase (TGT) catalytic subunit. Gene 265: 205–212. 11255023 10.1016/s0378-1119(01)00368-7

[37] Brenk R, Stubbs MT, Heine A, Reuter K, Klebe G (2003) Flexible adaptations in the structure of the tRNA-modifying enzyme tRNA-guanine transglycosylase and their implications for substrate selectivity, reaction mechanism and structure-based drug design. ChemBioChem 4: 1066–1077. 14523925 10.1002/cbic.200300644

[38] Gerber H-D, Klebe G (2012) Concise and efficient syntheses of preQ_1_, queuine and *ent*-queuine. Org Biomol Chem 10: 8660–8668. 23032613 10.1039/c2ob26387d

[39] Jerabek-Willemsen M, Wienken CJ, Braun D, Baaske P, Duhr S (2011) Molecular interaction studies using microscale thermophoresis. ASSAY and Drug Development Technologies 9: 342–353. 21812660 10.1089/adt.2011.0380 PMC3148787

[40] Kung FL, Garcia GA (1998) tRNA-guanine transglycosylase from *Escherichia coli*: recognition of full-length ‘queuine-cognate’ tRNAs. FEBS Lett 431: 427–432. 9714557 10.1016/s0014-5793(98)00801-1

[41] Sheriff S, Hendrickson WA, Smith JL (1987) Structure of myohemerythrin in the azidomet state at 1.7/1.3 Å resolution. J Mol Biol 197: 273–296. 3681996 10.1016/0022-2836(87)90124-0

[42] Thomas CE, Chen Y-C, Garcia GA (2011) Differential heterocyclic substrate recognition by, and pteridine inhibition of *E. coli* tRNA-guanine transglycosylase. Biochem Biophys Res Comm 410: 34–39. 21640076 10.1016/j.bbrc.2011.05.100 PMC3124622

[43] Lovell SC, Word JM, Richardson JS, Richardson DC (2000) The penultimate rotamer library. Proteins 40: 389–408. 10861930

[44] Boeckmann B, Bairoch A, Apweiler R, Blatter MC, Estreicher A, et al (2003) The SWISS-PROT protein knowledgebase and its supplement TrEMBL in 2003. Nucleic Acids Res 31: 365–370. 12520024 10.1093/nar/gkg095 PMC165542

[45] Chenna R, Sugawara H, Koike T, Lopez R, Gibson TJ, et al (2003) Multiple sequence alignment with the Clustal series of programs. Nucleic Acids Res 31: 3497–3500. 12824352 10.1093/nar/gkg500 PMC168907

[46] Eswar N, Marti-Renom MA, Webb B, Madhusudhan MS, Eranian D, et al (2000) Comparative protein structure modelling with MODELLER. Current Protocols in Bioinformatics Supplement 15: 5.6.1–5.6.30. 10.1002/0471250953.bi0506s15 PMC4186674 18428767

[47] Romier C, Ficner R, Reuter K, Suck D (1996) Purification, crystallization, and preliminary X-ray diffraction studies of tRNA-guanine transglycosylase from *Zymomonas mobilis* . Proteins 24: 516–519. 8860000 10.1002/(SICI)1097-0134(199604)24:4<516::AID-PROT11>3.0.CO;2-O

[48] Curnow AW, Kung FL, Koch KA, Garcia GA (1993) tRNA-guanine transglycosylase from *Escherichia coli*: gross tRNA structural requirements for recognition. Biochemistry 32: 5239–5246. 8494901 10.1021/bi00070a036

[49] Wienken CJ, Baaske P, Rothbauer U, Braun D, Duhr S (2010) Protein-binding assays in biological liquids using microscale thermophoresis. Nat Commun 1: 100. 20981028 10.1038/ncomms1093

[50] Otwinowski Z, Minor W (1997) Processing of X-ray diffraction data collected in oscillation mode. Methods Enzymol 276: 307–326. 10.1016/S0076-6879(97)76066-X 27754618

[51] Battye TGG, Kontogiannis L, Johnson O, Powell HR, Leslie AGW (2011) iMOSFLM: a new graphical interface for diffraction-image processing with MOSFLM. Acta Crystallogr Sect D 67: 271–281. 21460445 10.1107/S0907444910048675 PMC3069742

[52] Evans PR (2011) An introduction to data reduction: space-group determination, scaling and intensity statistics. Acta Crystallogr Sect D 67: 282–292. 21460446 10.1107/S090744491003982X PMC3069743

[53] Brunger AT, Adams PD, Clore GM, DeLano WL, Gros P, et al (1998) Crystallography and NMR system: A new software suite for macromolecular structure determination. Acta Crystallogr Sect D 54: 905–921. 9757107 10.1107/s0907444998003254

[54] Sheldrick GM, Schneider TR (1997) SHELXL: high-resolution refinement. Methods Enzymol 277b: 319–343. 18488315

[55] Adams PD, Afonine PV, Bunkóczi G, Chen VB, Davis IW, et al (2010) PHENIX: a comprehensive Python-based system for macromolecular structure solution. Acta Crystallogr Sect D 66: 213–221. 20124702 10.1107/S0907444909052925 PMC2815670

[56] Emsley P, Cowtan K (2004) Coot: model-building tools for molecular graphics. Acta Crystallogr Sect D 60: 2126–2132. 15572765 10.1107/S0907444904019158

[57] Winn MD, Ballard CC, Cowtan KD, Dodson EJ, Emsley P, et al (2011) Overview of the *CCP*4 suite and current developments. Acta Crystallogr Sect D 67: 235–242. 21460441 10.1107/S0907444910045749 PMC3069738

[58] Painter J, Merritt EA (2006) TLSMD web server for the generation of multi-group TLS models. J Appl Crystallogr 39: 109–111.

[59] Laskowski RA, MacArthur MW, Moss DS, Thornton JM (1993) PROCHECK: a program to check the stereochemical quality of protein structures. J Appl Crystallogr 26: 283–291.

[60] Kleywegt GJ, Zou JY, Kjeldgaard M, Jones TA, Around O (2001) In International Tables for Crystallography; Rossmann MG, Arnold E, Eds, Kluwer Academic Publishers: Dordrecht Vol F, 353–356.

