# Supplementary material for: Investigation of Specificity Determinants in Bacterial tRNA-Guanine Transglycosylase Reveals Queuine, the Substrate of Its Eucaryotic Counterpart, as Inhibitor
Source: PLoS One. 2013 May 21;8(5):e64240. doi: 10.1371/journal.pone.0064240 (PMC3660597; doi:10.1371/journal.pone.0064240)
Supplement: Table S1 — Overview of crystal structures analysed by Biela et al. (2013). (DOCX) [file pone.0064240.s004.docx]

## Table S1

Overview of crystal structures analysed in Biela *et al.* (2013)

| **Tgt variant · ligand** | **PDB ID** | **resolution** |
| --- | --- | --- |
| Tgt("WT") [37] | **1ozm** | 1.95 Å |
| Tgt("WT")·preQ_1_ [37] | **1ozq** | 1.90 Å |
| Tgt("WT")·Q |  | 1.21 Å |
| Tgt(Cys158Val) | **4gd0** | 1.29 Å |
| Tgt(Cys158Val)·guanine | **4h7z** | 1.68 Å |
| Tgt(Cys158Val)·preQ_1_ | **4e2v** | 1.18 Å |
| Tgt(Cys158Val)·Q | **4hvx** | 1.82 Å |
| Tgt(Val233Gly) | **3bl3** | 2.25 Å |
| Tgt(Val233Gly)·preQ_1_ | **3bld** | 1.19 Å |
| Tgt(Val233Gly)·Q | **4hsh** | 1.56 Å |
| Tgt(Cys158Val/Val233Gly) | **4h6e** | 1.42 Å |
| Tgt(Cys158Val/Val233Gly)·preQ_1_ | **4gcx** | 1.45 Å |
| Tgt(Cys158Val/Val233Gly)·Q | **4hqv** | 1.66 Å |
| Structures not discussed in detail: | | |
| Tgt(Cys158Val/Ala232Ser/Val233Gly) | **2nso** | 1.60 Å |
| Tgt(Cys158Val/Ala232Ser/Val233Gly)·preQ_1_ | **2nqz** | 1.46 Å |
| Tgt(Cys158Val/Ala232Ser/Val233Gly)·Q | **3blo** | 1.60 Å |
