# Supplementary material for: Investigation of Specificity Determinants in Bacterial tRNA-Guanine Transglycosylase Reveals Queuine, the Substrate of Its Eucaryotic Counterpart, as Inhibitor
Source: PLoS One. 2013 May 21;8(5):e64240. doi: 10.1371/journal.pone.0064240 (PMC3660597; doi:10.1371/journal.pone.0064240)
Supplement: Table S2 — Crystallographic data collection and refinement statistics of further structures related to the study of Biela et al. (2013). (DOCX) [file pone.0064240.s005.docx]

## Table S2

Crystallographic data collection and refinement statistics of further structures related to this study

| **Crystal data*** | Tgt(Cys158Val/Ala232Ser/Val233Gly) | Tgt(Cys158Val/Ala232Ser/Val233Gly)·  preQ_1_ | Tgt(Cys158Val/Ala232Ser/Val233Gly)·  Q | Tgt(Cys158Val/Ala232Ser/Val233Gly)  ·Boc-preQ_1_ |
| --- | --- | --- | --- | --- |
| **PDB ID** | **2nso** | **2nqz** | **3blo** | **3bll** |
| **A. Data Collection and Processing** | | | | |
| Beamline | BL 14.2 | BL 14.2 | BL 14.2 | BL 14.2 |
| λ (Å) | 0.91841 | 0.91841 | 0.91841 | 0.91841 |
| Space group | *C2* | *C2* | *C2* | *C2* |
| *a*, *b*, *c* (Å) | 90.7, 64.8, 70.8 | 89.9, 64.9, 71.2 | 90.4, 64.8, 70.3 | 90.0, 65.9, 70.5 |
| β (°) | 96.3 | 96.4 | 95.9 | 95.6 |
| Matthews coefficient (Å^3^/Da) | 2.4 | 2.4 | 2.4 | 2.4 |
| Solvent content (%) | 48.7 | 48.7 | 48.7 | 48.7 |
| **B Diffraction Data** | | | | |
| Resolution range*^a^* (Å) | 50-1.60 (1.63-1.60) | 20-1.46 (1.49-1.46) | 20-1.60 (1.63-1.60) | 20.-1.26 (1.29-1.26) |
| No. of unique reflections | 53,101 | 69,580 | 51,989 | 97,859 |
| Completeness*^a^* (%) | 98.6 (79.8) | 98.6 (80.7) | 97.5 (89.2) | 90.4 (63.3) |
| Redundancy | 2.9 | 3.2 | 2.4 | 3.4 |
| R(I)_sym_*^a,b^* (%) | 7.1 (34.5) | 6.3 (21.4) | 5.2 (29.9) | 3.7 (31.9) |
| I/σ(I) *^a^* | 15.0 (2.0) | 18.0 (3.9) | 18.0 (2.1) | 21.0 (2.2) |
| **C. Refinement** | | | | |
| Programm | ShelxL-97 | ShelxL-97 | ShelxL-97 | ShelxL-97 |
| *R*_work_*^c^* / *R*_free_*^d^* (%) | 16.2 / 22.1 | 15.0 / 19.8 | 18.0 / 21.3 | 14.9 / 18.2 |
| Protein residues | 364 | 361 | 349 | 347 |
| Water molecules | 185 | 296 | 183 | 245 |
| Ligand atoms | --- | 13 | 20 | 20 |
| Ramachandran plot | | | | |
| Residues in most favored regions (%) | 95.5 | 94.4 | 95.2 | 95.5 |
| Residues in additionally allowed regions (%) | 4.2 | 5.3 | 4.5 | 4.1 |
| Residues in generously allowed regions (%) | 0.3 | 0.3 | 0.3 | 0.3 |
| Mean *B*-factors (Å^2^) | | | | |
| Protein | 24.2 | 20.1 | 24.1 | 18.1 |
| Water | 30.7 | 30.8 | 30.8 | 28.9 |
| Ligand | --- | 22.6 | 39.9 | 28.9 |
| RMSD from ideality | | | | |
| rmsd angle (°) | 2.0 | 2.1 | 2.2 | 2.2 |
| rmsd bond (Å) | 0.009 | 0.011 | 0.010 | 0.012 |

* all mutated *Z. mobilis* Tgt variants investigated in this study contain an additional Tyr106Phe mutation (see main text).

*^a^*) number in parentheses is for highest resolution shell

*^b^*) , with *I* representing the observed intensity and *Ī* representing the average intensities for multiple measurements.

*^c^*) $R_{work}=\frac{\sum_{hkl} \left|F_{obs}-F_{calc}\right|}{\sum_{hkl} \left|F_{obs}\right|}.$

*^d^*) *R*_free_ was calculated as *R*_work_ but on 5% of the data excluded from the refinement.
